# A non-classical route of efficient plant uptake verified with fluorescent nanoparticles and root adhesion forces investigated using AFM

**DOI:** 10.1038/s41598-020-75685-3

**Published:** 2020-11-06

**Authors:** Sandeep Sharma, Mohd. Muddassir, Saraladevi Muthusamy, Pardeep Kumar Vaishnav, Manish Singh, Deepak Sharma, Selvaraju Kanagarajan, Vijayakumar Shanmugam

**Affiliations:** 1 Institute of Nano Science and Technology, Habitat Centre, Phase- 10, Sector- 64, Mohali, Punjab 160062 India; 2 CSIR-Institute of Microbial Technology, Chandigarh, India; 3 Applied Microbiology, Department of Chemistry, Lund University, Lund, Sweden; 4 Microscopy Division, AIIMS, New Delhi, India; 5 Department of Plant Breeding, Swedish University of Agricultural Sciences, Alnarp, Sweden

**Keywords:** Plant sciences, Nanoscience and technology

## Abstract

Classical plant uptake is limited to hydrophilic or water-dispersible material. Therefore, in order to test the uptake behaviour of hydrophobic particles, here, we tested the fate of hydrophobic particles (oleylamine coated Cu_2-x_Se NPs (CS@OA)) in comparison to hydrophilic particles (chitosan-coated Cu_2-x_Se NPs (CS@CH)) by treatment on the plant roots. Surprisingly, hydrophobic CS@OA NPs have been found to be ~ 1.3 times more efficient than hydrophilic CS@CH NPs in tomato plant root penetration. An atomic force microscopy (AFM) adhesion force experiment confirms that hydrophobic NPs experience non-spontaneous yet energetically favorable root trapping and penetration. Further, a relative difference in the hydrophobic vs. hydrophilic NPs movement from roots to shoots has been observed and found related to the change in protein corona as identified by two dimensional-polyacrylamide gel electrophoresis (2D-PAGE) analysis. Finally, the toxicity assays at the give concentration showed that Cu_2-x_Se NPs lead to non-significant toxicity as compared to control. This technology may find an advantage in fertilizer application.

## Introduction

Plants have evolved slowly through natural selection processes, which have been rapidly increased by biotechnology for human requirements^1^. Recently, in phytonanotechnology, plants have been tuned positively by the intrinsic properties of nanoparticles (NPs), such as electron conductivity (improved the electron transport rate of photosystem 1 by 8.8%)^2^, ROS scavenging^3^, water/nutrient retention/supply^4^, and genetic manipulation^5,6^. Furthermore, NPs loaded with the chemical active ingredients have also extended the scope of microsurgery in plants through triggered release^7–10^. To understand the physiological consequences^11–14^, a comparison of the vascular uptake of NPs was carried out. Interestingly, some aquatic plants showed greater uptake of NPs than ions^15^.

Classical plant uptake mechanism includes passive spontaneous diffusion, mass flow, ion exchange, and active energy-intensive carrier-assisted method^16^. In NPs uptake also, similar passive mechanisms were identified in metals (M)^17,18^, metal oxides (MO)^19,20^, chalcogenides (MS)^21^, and carbon materials^2,5,22^. Even carrier-mediated transport of NPs within the plant cell to different organelles were also documented^23^. These penetrating NPs were found to be transported both by symplastic and apoplastic modes and have shown xylem and phloem transport^24^. Furthermore, the role of NPs coating, such as with/without citrate^25^, and surface charges^26,27^ on plant uptake behavior was also studied.

For efficient plant-gene manipulation with NPs, forced-injection strategies were developed by us and others, but such pressure-assisted delivery systems are not easy to adopt in large-scale field applications^28,29^. Hence, we envisage that a similar forced injection method in a large-scale may be feasible by having hydrophobic surface modification. Both theoretically and experimentally, hydrophobicity in combination with mildly hydrophilic groups was found to show excellent adhesion of water drop even with a tilt of 180°^30–34^. Similarly, hydrophobic NPs were found to be more easily penetrate lipid membranes in aqueous media^35^. This enhanced adhesion by hydrophobicity, motivated us to test the plant uptake efficiency between hydrophobic vs. hydrophilic NPs. Recently, many statistical surveys have reported the global value of vertical farming in 2018 as $2.23 billion and in 2026 it is expected to be $12.77 billion, which emphasizes the practical importance of this study for agro-industry.

For this comparison, intensely fluorescent Cu_2-X_Se NPs were used, which are non-toxic, unlike cadmium particles. In optical NPs assisted bio-tracking, generally cadmium-based chalcogenide like cadmium sulphides and cadmium selenide have been used and appreciated for the intense fluorescence in most of the publication. In place of cadmium-based material, copper-based materials are more biocompatible, hence copper selenide is chosen. Owing to the antioxidant role of selenium based amino acid^36^, and the recommendation of copper and selenium as plants micronutrient fairly convince us to use Cu_2-X_Se NPs as the model particle^37^. Furthermore, Cu_2-X_Se is an isoform of the stable sulphides chalcogenide-family antidote that is preferentially formed by plants to overcome metal ion and metal oxide toxicity^38–40^. Here, the Cu_2-X_Se NPs are synthesized in oleylamine (CS@OA) as reported before, which gives them hydrophobicity. To have an equivalent hydrophilic particle, the as-prepared Cu_2-X_Se NPs were coated with amphiphilic biopolymer chitosan (CS@CH) and transferred into hydrophilic^41–43^. Synthesizing and stabilizing NPs in solution without capping agent is nearly impossible, which avoids question compare with naked NPs^44^. To test the uptake kinetics, an economically valuable model plant physiology plant i.e., tomato was used^45,46^. Additionally, to our knowledge for the first time, force-distance measurements has been conducted using AFM with the NPs modified tips against a root to understand the adhesion dependence.

## Results and discussion

### Synthesis and characterization

**Figure 1:**
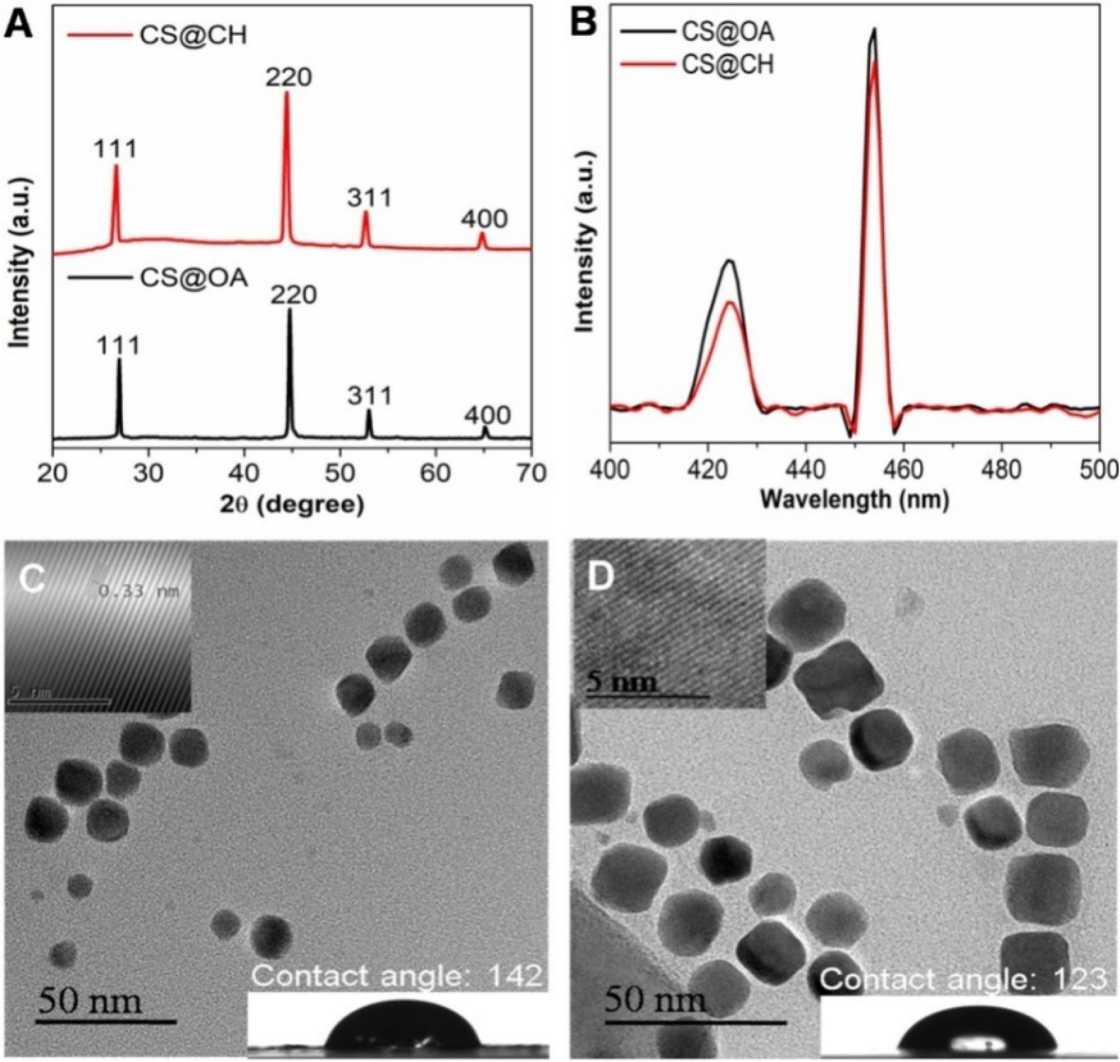
Characterization of the Cu_2-x_Se NPs (as-prepared oleylamine-coated CS@OA and chitosan-coated CS@CH). (**A**) XRD patterns of CS@OA (black curve) and CS@CH NPs (red curve). (**B**) PL spectra of CS@OA (black curve) and CS@CH (red curve) recorded at a 370 nm excitation wavelength. (**C**,**D**) TEM images of CS@OA and CS@CH showing the Cu_2-x_Se NPs, respectively (inset (top left corner): HR-TEM images of the CS@OA and CS@CH NPs showing a 0.33 nm lattice spacing, corresponding to the (111) plane of Cu_2-x_Se) (inset (bottom right corner): contact angle on glass substrates coated with CS@OA or CS@CH NPs).

To study the effects of NPs surface polarity on plant uptake, as-prepared hydrophobic Cu_2-x_Se NPs with oleylamine coating and hydrophilic Cu_2-x_Se NPs with chitosan coating were tested against the model plant viz., tomato. The X-ray diffraction (XRD) pattern of the as-prepared oleylamine coated Cu_2-x_Se NPs (Fig. 1A) shows diffraction peaks at 26.77, 44.72, 53.03, and 65.27° that match the (111), (220), (311), and (400) planes of face-centred-cubic Cu_2-x_Se (JCPDS 06-0680) (for brevity, this material will be denoted as CS@OA).

Following the XRD confirmation of the as-prepared Cu_2-x_Se NPs, to develop hydrophilic substitutes of the same, Cu_2-x_Se NPs were coated with chitosan (for brevity, this material will be denoted as CS@CH). The chitosan coating was difficult to confirm by Fourier transform infrared (FT-IR) spectrophotometer, as the oleylamine signals overlap with the chitosan signals w.r.t N–H bending at 1381 cm^-1^ and 1625 cm^−1^ and C–N bonding/N–H stretching at 3400 cm^−1^ (Fig. S1)^47–49^. However, the visual observation of the CS@CH NPs dispersed well in distilled water compared to the complete precipitation of the CS@OA NPs in distilled water confirms the coating (Fig. S2). Furthermore, to quantify the change in the NPs surface polarity, the material before and after chitosan coating was drop cast onto glass, and the contact angle was measured. The contact angle of the CS@OA NPs has been found to be 142° (this is close to the value of superhydrophobicity (150°)^50^, which after chitosan coating in CS@CH NPs decreased to 123° (inset in Fig. 1C,D). This chitosan coating has not been found to affect the absorbance intensity (Fig. S3). The intense fluorescence of the Cu_2-x_Se NPs needed to be retained for the tracking of the NPs through confocal imaging. Hence, the photoluminescence (PL) spectra of CS@OA NPs and CS@CH NPs were measured in 1:1 ratio of ethanol: water mixture at 370 nm excitation wavelength. The spectra show no major compromises in signal intensity after coating, which ensures its insignificance on the imaging (Fig. 1B). The transmission electron microscope (TEM) image of the CS@OA NPs shows monodisperse NPs with a size distribution of approximately 15 ± 8 nm (Fig. 1C). The high resolution (HR) TEM (HR-TEM) (inset in Fig. 1C) shows a lattice spacing of 0.33 nm, which corresponds to the (111) plane of Cu_2-x_Se NPs. The TEM image after chitosan coating shows that the size and shape of the NPs is stable (Fig. 1D). The size of CS@CH NPs has been found in the range from 15 to 30 nm. In agreement with the higher molecular weight of chitosan, the hydrodynamic peak size of the CS@CH NPs (PDI = 0.123) has been found to be 9 nm greater than that of the CS@OA (PDI = 0.087) NPs (Fig. S4). The calculated number of particles were 2.5 × 10^13^/mL.

### Uptake study

**Figure 2:**
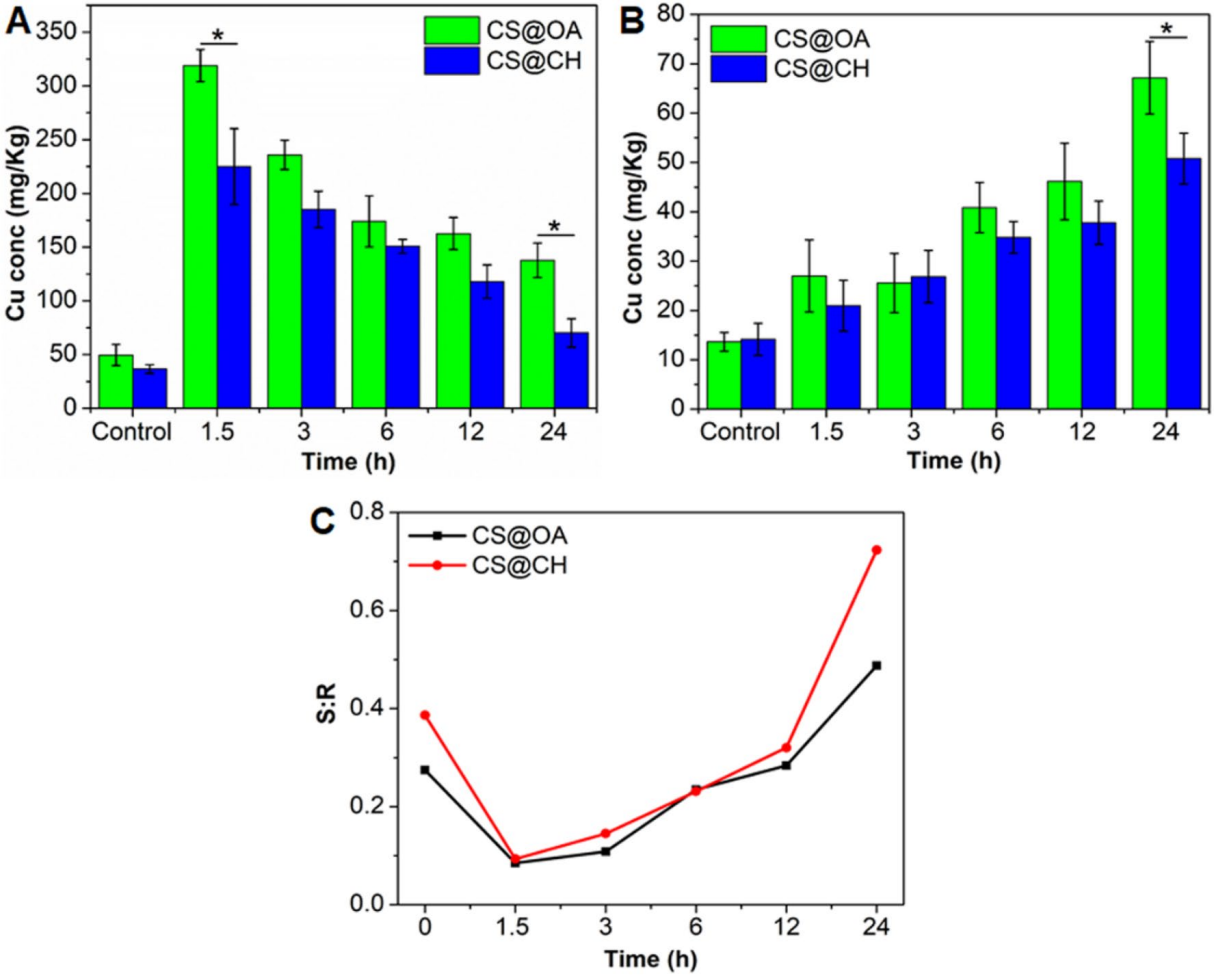
(**A**,**B**) Copper accumulation in the roots (**A**) and shoots (**B**) at different incubation times after spraying the roots with either CS@OA (treated group 1) or CS@CH (treated group 2) NPs estimated with ICP-MS (the copper concentration is normalized to per kg of root and shoot dry weight). (**C**) Shoot: root copper content ratio.

Following the material characterization, both CS@OA and CS@CH NPs were sprayed onto the roots of 30-day-old plants. One group was sprayed with CS@OA NPs and the other group was sprayed with CS@CH NPs. After brief air drying, the plants were incubated in the hydroponic medium. The NPs that had not landed on the roots were collected on a glass backspot and estimated to be ~ 200 µg. The root and shoot samples were collected at 1.5, 3, 6, 12, and 24 h intervals, oven-dried and then quantified with inductively coupled plasma mass spectrometry (ICP-MS) after microwave acid digestion (Fig. 2A,B). Before oven drying and digestion steps, the root biomass of samples collected after every time intervals were washed with 0.1 M HNO_3_, to remove the NPs just adhered without uptake by the root^51^. The scanning electron microscope (SEM) images of the unwashed roots (Fig. S5) and after washing with 0.1 M HNO_3_ (Fig. S6) confirms that the adhered particles were removed. The uptake study (Fig. 2A) clearly shows that the hydrophobic CS@OA NPs have the ability to quickly enter into the roots and are taken up at a rate that is ~ 1.3 times the uptake rate of the hydrophilic CS@CH NPs after the initial 1.5 h incubation with the root.

**Figure 3:**
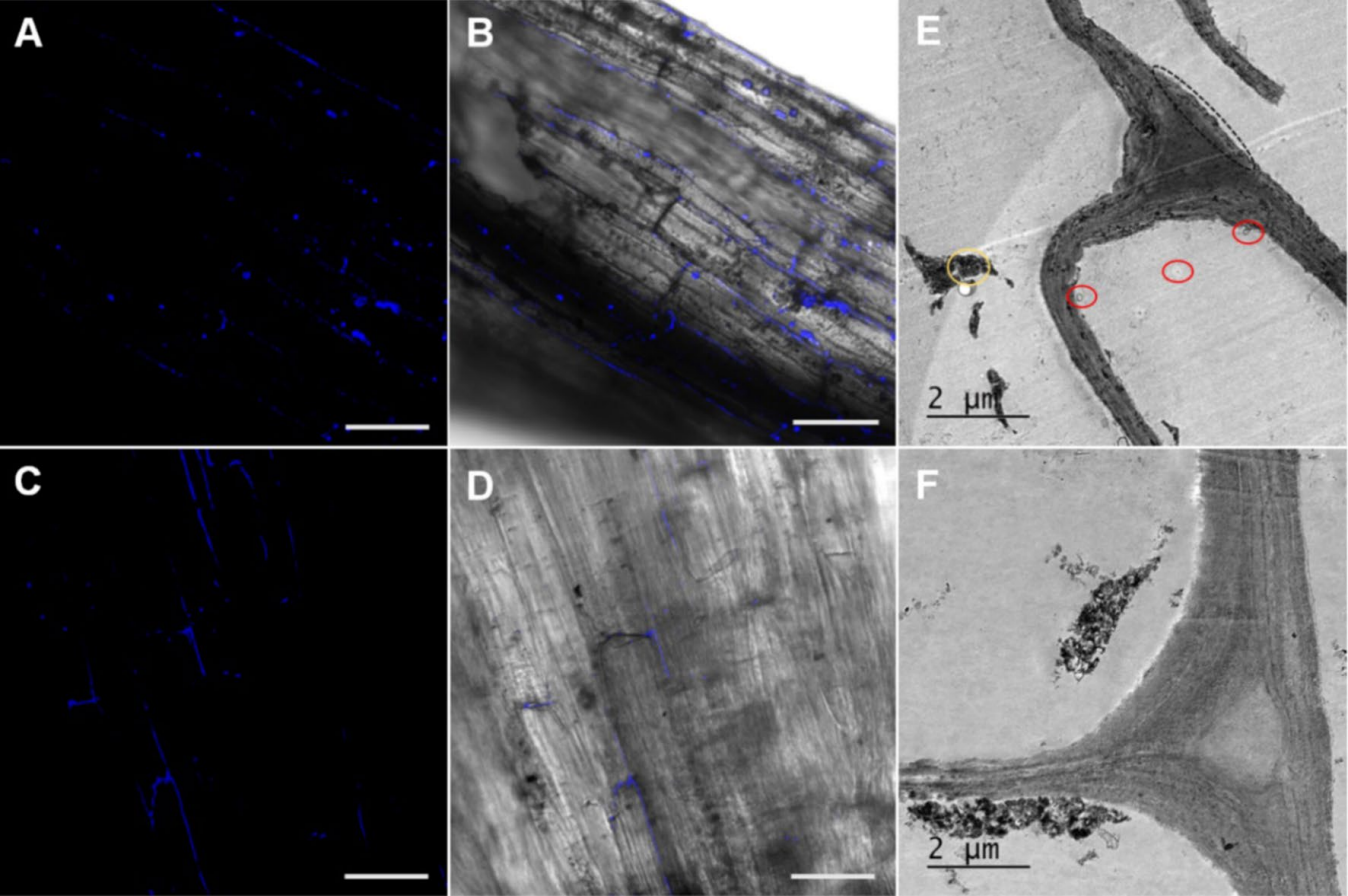
Confocal and TEM images of tomato plant tissue showing NPs uptake. (**A**–**D**) The confocal images of tomato roots sprayed with CS@OA (**A**,**B**) and CS@CH (**C**,**D**) NPs after 1 h of incubation time merged with the brightfield image (blue colour dots indicates the NPs) (scale bar is 20 µM). (**E**,**F**) TEM images of the microtome sections of tomato roots sprayed with CS@OA (**E**) and CS@CH (**F**) after 3 h of incubation time. **A**,**B**,**E** corresponds to treated group 1 and **C**,**D**,**F** corresponds to treated group 2.

The uptake concentration started to decrease gradually in the root, unlike previous studies where a continuous increase in the NPs content in the root was observed^52–54^. This is obviously because of the (1) lack of continuous NPs supply from the medium, unlike in previous studies, (2) transport to the shoot from the root (Fig. 2B), and (3) possible restriction of any particles adhered to the root. Despite the unavailability of additional NPs in the incubation medium, the uptake in the roots has been found to be more than that in the shoot at any given time point in the 24 h observation period. This may be due to an inhibition of the NPs movement, which is in agreement with the previously reported long observation times for different hydrophilic NPs compared to that of ions^20,55,56^. Restriction of movement across the tissue in hydrophilic CeO_2_ particles resulted in their dissolution into ions after 4 days^19^. Interestingly, the ratio of the NPs movement to shoots from the roots have been found higher in the treatment sprayed with hydrophilic NPs (0.72) than in the hydrophobic NPs (0.48) (Fig. 2C). To negate the role of ethanol in uptake, we test the uptake of CS@CH NPs at 1.5 h, after pure ethanol spraying followed by CS@CH NPs spraying in water. The uptake of CS@CH NPs (212 ± 8 mg/kg) found to be a little lesser or almost equal to the treatment without ethanol pre-spraying, which clearly negates any role of ethanol. We also quantified the leaching of Cu ions from CS@OA and CS@CH NPs in the medium by ICP-MS after 24 h of incubation and found ~ 1.5% copper in CS@OA and < 1.5% in CS@CH NPs, which is insignificant to influence the treatment.

The intense fluorescence from the Cu_2-X_Se NPs allowed the NPs to be tracked in the plant tissue; here, an image taken from a root after 1 h of incubation using confocal laser scanning microscopy (CLSM) is given in Fig. 3A–D. This study confirms that the uptake occurred as intact NPs in the plant tissue rather than by dissolution or oxidation of the NPs to ions. The CLSM image of the untreated root sample reveals the absence of particles (Fig. S7). The 3D CLSM images of the tissues treated with CS@OA and CS@CH NPs are given in videos 1 and 2, respectively. This was further confirmed by the TEM micrographs of the microtome sections of the roots after 3 h of incubation (vide infra).

Following the CLSM study, the microtome sections of the roots were stained and observed by TEM. In the TEM images, the darkly contrasted CS@CH and CS@OA NPs shows differences in their patterns of particle aggregation, distribution, and transport in the tissue. The hydrophobic CS@OA NPs have been predominantly present in the intercellular region and showed similarly restricted movement as that observed for the less hydrophilic pristine carbon nanotubes in plant cells; however, in that study the polarity wasn’t discussed^57^ (Fig. 3E). CS@OA NPs aggregation in the intercellular space may be due to cell wall lipid assisted liposome formation and may eventually restrict its intracellular movement. Within the intercellular area, a major portion of the hydrophobic CS@OA NPs have been found to be aligned in the bilayer cell membranes unexposed to the polar heads (encircled in black gradient lines). The element composition of these particles was confirmed with point EDX elemental analysis (Fig. S8); the nickel signal observed is from the grid, since nickel grid was used in place of copper grid to avoid copper signal overlap and lead signal observed is from the staining agent. In the hydrophobic CS@OA NPs-treated plant, many endocytosis-like bodies have been observed (encircled in red), which once again supports lipid-covered body formation in the hydrophobic treatment. Supporting this claim, hydrophobic NPs were reported to easily form liposomes through bilayer disruption^35^.

In contrast, the hydrophilic CS@CH NPs have been found predominantly distributed in the intracellular region, which may be due to the ability of the hydrophilic NPs to interact with the polar head groups of the cell membrane (Fig. 3F). However, this does not negate the possibility of CS@CH NPs movement in the intercellular region. A closer look at the gradient circle shows NPs-aligned movement without aggregation. Both CS@OA and CS@CH NPs did occupy the intercellular gas space. The predominant intracellular distribution of the CS@CH NPs and their well distributed (without aggregation) intercellular presence may be due to their compatibility with an aqueous medium. Unlike hydrophobic NPs, in CS@CH NPs treatment, endocytotic bodies have not been observed which may be due to their ability to directly enter the cell. Similar direct entry without endocytosis was observed in plant protoplast cells^58^. The particle size distribution in the intercellular space is given in Fig. S9 (the microtome location of the NPs is given just above the graph), which confirms the stability of the NPs in the plant tissue. However, over time, they may not be stable due to the enzymatic action of the plant, especially in the leaves, and over time, the particles may dissociate into ions, as previously observed by radioactive signals^19^. The untreated root sample did not show the presence of any particles (Fig. S10).

### Mechanism of uptake

#### AFM adhesion force measurements

The enhanced penetration of CS@OA NPs into the root may be due to their binding, which motivated us to measure the force *vs.* distance between the root and the NPs in water using AFM. AFM silicon nitride tips were modified with CS@OA and CS@CH, separately, by incubation in the respective solutions for 5 h followed by sequential washing with water and ethanol. A fresh root tip was fixed onto a glass plate with a resin that could dry fast without any cross-reactions in order to keep the root alive^30,59,60^. Following this, water was added carefully with a syringe, and the AFM tip was placed in contact with the root with the assistance of the microscope.

**Figure 4:**
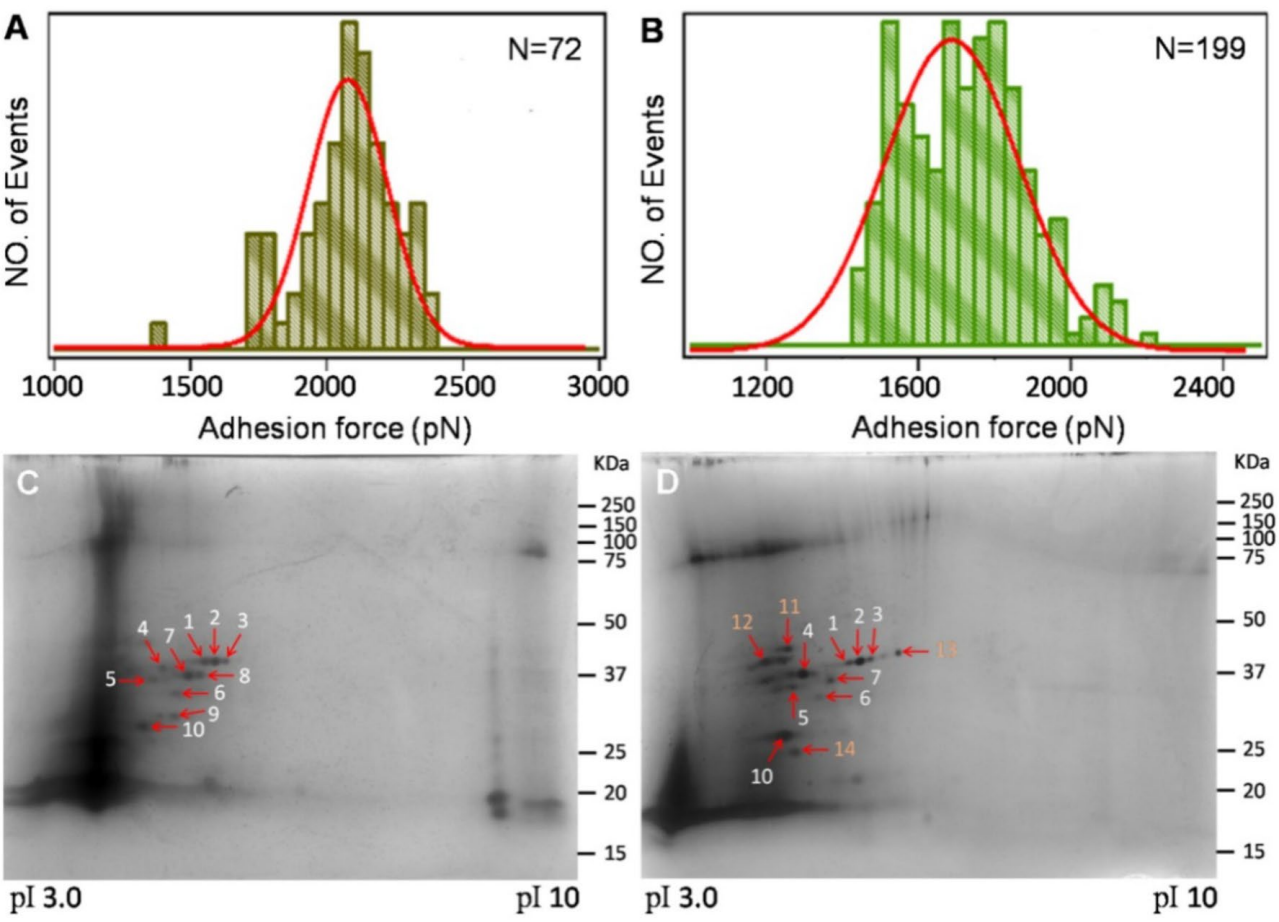
(**A**,**B**) Adhesion force between the root and an AFM tip modified with either CS@OA (A) or CS@CH (B) NPs in water. (**C**,**D**) 2D gel pattern of the protein corona extracted from roots with CS@OA (**C**) and CS@CH NPs (**D**) in a pH gradient of 3–10 with silver staining. **A**,**C** corresponds to treated group 1 and **B**,**D** corresponds to treated group 2.

The maximum from the Gaussian fitting of the adhesion force experienced by the CS@OA NPs has been found to be ~ 500 pN more than the force experienced by the CS@CH NPs (Fig. 4A,B). This adhesion could be driven by three reasons: (1) water, which pushes the hydrophobic NPs towards a solid substrate (here the root) or the hydrophobic particles unwillingness to allow water into the root/particle interspaces as confirmed with contact angle, (2) manipulation of the contacted hydrophobic NPs surface to become sticky with the ions present on the root surface as confirmed with AFM study^35^, and (3) as mentioned above, the ability of the hydrophobic NPs to penetrate the lipid surface as confirmed with ICP-MS^35^. Thus, active uptake where the energy contribution comes from the NPs, unlike classical active transport where the energy is expended by the plants, is identified. Thus, the adhesion force expressed by the NPs corroborates to the root uptake proportionally, similar proportional uptake was also reported earlier in the animal cells^61^. The maximum adhesion force expressed by the hydrophobic NPs have led to the enhanced root uptake.

#### Protein corona study

The curiosity to understand the ability of the hydrophilic NPs to show a greater root to shoot transport ratio motivated us to study its protein corona in comparison to that of the hydrophobic NPs. Protein coronae, which changes with the surface often decide the fate of NPs in animals^62–67^; which has been ignored in plants, is studied here by 2D-polyacrylamide gel electrophoresis (2D-PAGE). The intact NPs from the plant tissues were obtained by enzyme-assisted extraction following the protocol standardized by Dan et al^17^. In 2D gels, protein spots have been found to be distributed within a molecular mass range of 19–43 kDa and covering the pH range of 4.3–9.6. For the CS@OA NPs, 10 protein spots have been detected, while 14 protein spots have been detected for the CS@CH NPs. In the CS@OA sample, 2 proteins have been found down-regulated (numbers 4 and 10), and 4 protein spots have not been detected (numbers 11, 12, 13 and 14) compared to the proteins detected in the CS@CH sample (Fig. 4C,D). Interestingly, all of these protein spots (except 13) have been observed at acidic pH. To explain this pattern, close approximations of foreign body movements in roots viz., a mycorrhizal association report were compared. A similar downregulation of an acidic pI membrane protein was observed in tomato by mycorrhizal association^68^, which may be the adaptation strategy of the plant to restrict them in the root zone. Additionally, the presence of a few spots in the higher pI range for the CS@OA sample are poorly separated due to the poor protein solubility and contaminants in the buffer, which interfered with staining. Therefore, they may not be considered protein spots.

#### Toxicity study

**Figure 5:**
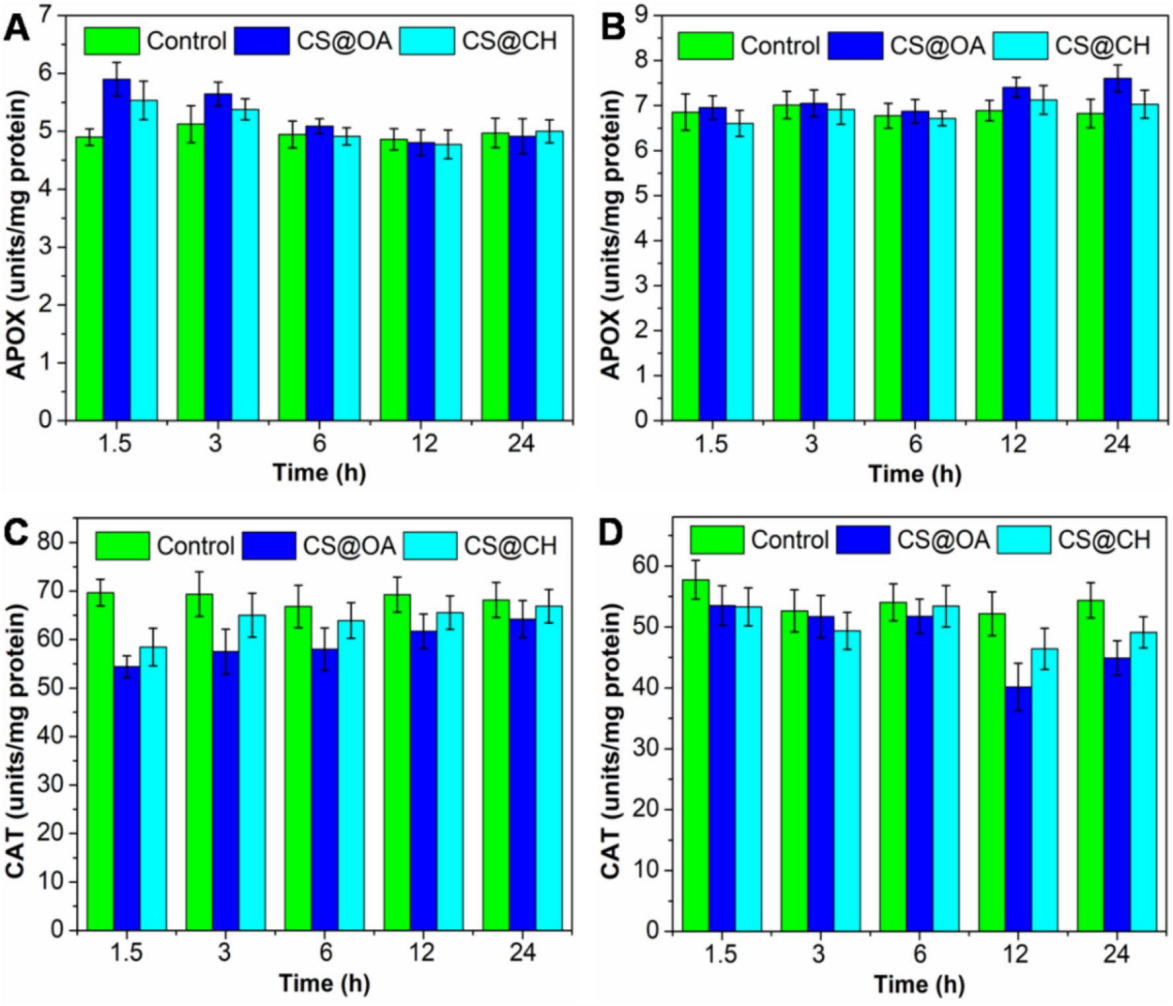
APOX and CAT antioxidant activity in tomato roots (**A**,**C**) and shoots (**B**,**D**) after exposure to Cu_2-x_Se (CS@OA and CS@CH) NPs compared to the control plants at different time points. Blue bars correspond to treated group 1, cyan bars correspond to treated group 2 and green bars correspond to the untreated group.

Finally, after elucidating the uptake mechanism, the toxicities of the hydrophobic and hydrophilic NPs were compared by using the ascorbate peroxidase (APOX) and catalase (CAT) activity assays. In roots, the APOX activity has been found to be increase after incubation with the NPs, especially at 1.5 h (Fig. 5A). The initial stress due to the plant uptake of foreign bodies may have resulted in the synthesis of H_2_O_2,_ whose enzymatic conversion may have raised the APOX activity^51^. Interestingly, with increasing incubation time, the activity has been found to be reduced, possibly because the plant adapted to the initial NPs load and because there is no further accumulation. In the shoots, the APOX activity has been found to be increase after 12 h of incubation, which may have been due to the time needed for a threshold amount of NPs accumulation to trigger APOX in the shoot (Fig. 5B). In case of the CAT activity, the reduction in the activity has been noted (Fig. 5C,D), which is contrary to the previous studies on metal NPs uptake^69^. There are few studies where a reduction in the CAT activity was also documented in the presence of an overexpression of the APOX activity, for instance here APOX is overexpressed, which can control oxidative stress^22,51^. Visual observation of the plants over 3 days surprisingly shows that the plants incubated after being sprayed with CS@OA NPs found to healthier than the plants incubated after being sprayed with CS@CH NPs. Further, the MTT assay was also performed to evaluate the toxicity caused by the CS@OA and CS@CH NPs. The assay shows the biocompatibility of the NPs because the percent root viability has been found to be > 90% after 24 h of treatment with CS@OA and CS@CH NPs (Fig. S11). Thus, at a 100 µg/plant concentration, no visual effect on plants incubated with hydrophobic NPs. Apart from the NPs concentration; there is a fair chance that trace amounts of selenium ions could have leached from the particles and enhanced the antioxidant/photosynthetic activity and photo-oxidative stress control^70–74^.

## Conclusions

Previous experience regarding NPs uptake has confirmed that uptake is a genus, species, variety, material, age, concentration, and size-dependent. However, there were no reports on the effects of NPs with hydrophobic surfaces, which are explained here. The enhanced uptake of hydrophobic NPs by the roots proves that non-classical forced penetration is more efficient. Hence, this enhanced uptake and sedentary behavior of hydrophobic NPs in the root can be adopted for eco-friendly leach-proof fertilizer application. This report also serves as an early warning to avoid exposing undesired hydrophobic NPs to edible plants in the context of enhanced phytoaccumulation. Furthermore, TEM images taken at the early incubation period reveal a predominance of hydrophobic NPs in the membrane bilayer, which has the future potential for spatial targeting in plants.

## Methods

### Materials

Ethanol, methanol, chloroform, glacial acetic acid, HNO_3_, H_2_O_2_, Na_2_HPO_4_, NaH_2_PO_4_, AgNO_3_, Na_2_CO_3_ and sodium dodecyl sulphate (SDS) were purchased from Merck, Bengaluru, India. Bovine serum albumin and chitosan powder were purchased from Sisco Research Laboratories, Chandigarh, India. Glutaraldehyde was purchased from TCI Chemicals, Chandigarh, India. A Spurr resin kit and OsO_4_ were purchased from Electron Microscopy Sciences, Delhi, India. Uranyl acetate was purchased from LobaChemie, India. CuCl, selenourea, oleylamine, citric acid, sodium citrate monobasic, lead citrate, Laemmli buffer, ammonium persulfate, trizma base, tetramethylethylenediamine (TEMED), acrylamide, *N,N*′- methylenebis(acrylamide), 3-(4,5-dimethythiazol-2-yl)-2,5-diphenyl tetrazolium bromide (MTT), KOH, phenylmethylsulfonyl fluoride (PMSF) cocktail, KH_2_PO_4_, K_2_HPO_4_, glycerol, and formaldehyde were purchased from Sigma-Aldrich, Bengaluru, India. Bradford reagent, macerozyme R-10, 1X phosphate buffer saline (PBS), sodium thiosulfate, and ascorbate were purchased from HiMedia, Mumbai, India. Rehydration buffer, IPG strips of pH range 3–10, a ReadyPrep 2D starter kit equilibration buffer I and II, and a ReadyPrep 2D clean-up kit was purchased from Bio-Rad, Gurugram, India. A precision plus protein kaleidoscope prestained protein ladder was purchased from Bio-Rad.

### Synthesis of Cu_2-x_Se NPs (CS@OA)

The arrested precipitation method was adopted for the synthesis of Cu_2-x_Se NPs with minor modifications^75^. In brief, a nitrogen-filled glove box (< 0.5 ppm oxygen) was employed for the preparation of the precursor mixtures. First, copper and selenium reactant mixtures were individually prepared and allowed to react simultaneously by hot injection. The copper reactant was prepared by the addition of 10 mL of oleylamine to 0.198 g of cuprous chloride in a round-bottom flask. Then, the mixture was heated under a nitrogen environment to 130 °C for 15 min along with stirring using a stir bar. The solution was cooled to 100 °C before the injection of selenium. The selenium reactant was prepared by the addition of 1 mL of oleylamine to 0.123 g of selenourea in a round-bottom flask. Then, the mixture was heated under nitrogen conditions to 200 °C for 15 min with stirring. Selenourea and oleylamine were injected into a flask containing cuprous chloride and oleylamine after being cooled to 160 °C. After injection, the solution appeared black and was further heated to 240 °C for half an hour. Then, the solution was allowed to cool to room temperature. The synthesized CS@OA NPs were precipitated by adding 10 mL of ethanol and washed 5 times with a chloroform-ethanol mixture with a ratio of 1:2 and finally dried under reduced pressure of 65 cm Hg.

### Synthesis of chitosan-coated Cu_2-x_Se NPs (CS@CH)

The synthesized CS@OA NPs powder (10 mg) was dispersed in 10 mL of a chitosan solution by bath sonication for 30 min. The chitosan solution was prepared by adding 50 mg of chitosan powder to 10 mL of distilled water at pH 4.5 under stirring. The pH of the distilled water was adjusted by glacial acetic acid^76^. After dispersion, the solution was centrifuged at 12,000 rpm for 10 min to remove unbound free polymer, followed by washing with distilled water. Finally, the chitosan-coated CS@OA NPs were dispersed in distilled water.

### Characterization of CS@OA and CS@CH NPs

A Bruker D8 Advance Diffractometer with a Cu Kα_1_ radiation source (λ = 1.5406 Å) was used for XRD pattern analysis at 40 kV and 25 mA. A Cary 600 Series FT-IR spectrophotometer (Agilent Technologies) was used to record the FT-IR spectra. The contact angle was measured by DIGIDROP modular contact angle technology and analyzed by Visio drop software. For this study, CS@OA and CS@CH NPs samples were dispersed in ethanol and distilled water, respectively, and a film was prepared by coating the samples onto a glass substrate surface. The glass substrate and other surfaces were cleaned with acetone and ethanol using a cotton swab, dried with a clean cotton swab, and then placed onto the sample stage. The contact angle was measured by gently depositing a drop of deionized water onto the film on the substrate surface using a microsyringe at ambient temperature. The contact angle was measured instantly after contact of the water drop with the NPs film. Contact angle was calculated by the computer software in the goniometer without the operator intervention. UV–Visible absorption spectra were recorded by a UV–Visible spectrophotometer (UV-2600, Shimadzu). An Edinburgh Instruments was used to record the PL emission spectra at 370 nm excitation wavelength. For UV–Visible and PL analysis, CS@OA and CS@CH powders were dispersed in an ethanol–water mixture with a ratio of 1:1 at a concentration of 300 ppm. A JEOL JEM-2100 microscope was used for TEM and HR-TEM analysis at 200 kV. The samples were prepared by dropping 7 μL of a highly diluted and dispersed sample solution onto a carbon-coated copper grid and wicking off excess solution after 1 min with filter paper. The size distribution on TEM images was calculated by using Image-J software. A Malvern Zetasizer Nano ZSP Instrument was used to measure the hydrodynamic diameter of each sample at 25 °C. Well-dispersed samples of 50 ppm concentration was prepared for the hydrodynamic diameter measurements were added into a clear glass dynamic light scattering (DLS) cuvette. In parameters, total three runs were set for each sample, each run was for 2 min and the equilibration time was set 120 s.

### Instrumentation in determining NPs and plant interaction

The copper concentrations in root tissues were quantified by using ICP-MS (Agilent 7700 series). CLSM (Carl Zeiss microscope LSM 800) was used to locate the CS@OA and CS@CH NPs in the root cells. CLSM images were processed using ImageJ software. AFM experiments were carried out on a commercial AFM instrument (Force Robot 00574, JPK Instruments, Berlin, Germany). The force-distance curves were recorded by commercial software from JPK and analyzed by custom-written procedures in Igor Pro 6.2 (Wavemetrics, Inc.). For the experiment, CS@OA and CS@CH NPs were dispersed in ethanol and water, respectively, at a concentration of 300 ppm. The PROTEAN i12 IEF system (Bio-Rad) was used for the separation of proteins in the first dimension, and the Mini-PROTEAN system (Bio-Rad) was used for the separation of proteins in the second dimension. Gel pictures were captured by using a gel doc (Bio-Rad) with Image Lab software.

### Uptake study of CS@OA and CS@CH NPs

#### Plant treatment

As characterized particles were applied aeroponically on the tomato roots following the standard procedure^77,78^. For this study, 30-day-old tomato plants were collected from the green house and sorted in such a way that they have approximately similar biometric parameters (9–10 cm, and the root lengths were 1.5–2 cm) and divided into 2 groups. The as-prepared hydrophobic CS@OA NPs were dispersed in ethanol, and the hydrophilic CS@CH NPs were dispersed in distilled water for spraying. First group of plants was sprayed with the CS@OA NPs, and the second group was sprayed with CS@CH NPs, dispersed in 1 mL respective solvent at 300 ppm concentration. After briefly air drying, the plants were incubated in the 20 mL hydroponic medium for 1.5, 3, 6, 12, and 24 h, following which the samples were oven-dried and then quantified with ICP-MS after microwave acid digestion. The NPs that had not landed on the roots were collected on a glass backstop from two samples per treatment, digested, and analyzed with the ICP-MS. Details of the ICP-MS protocol is given below.

#### ICP-MS analysis

After the specified time period, the plants were collected and washed with 0.01 M HNO_3_ and Milli-Q water to remove any adhered particles that had not entered the roots. Then, the plants were sectioned into roots and stems (cut 5 cm from the shoot start point) and dried properly at 60 °C in an oven, and weighed. The dry weights of the 50 mg roots and 150 mg shoots were taken for the metal content analysis. The treated samples were then digested by using 1 mL of metal-grade HNO_3_ and H_2_O_2_ (1:4) as described previously^51^. Then, the samples were diluted 13-fold in distilled water and analyzed using ICP-MS. Further, to quantify the leaching of copper ions from CS@OA or CS@CH NPs, the medium in which the plant was sprayed and incubated for 24 h, was collected. This medium was centrifuged at 10,000 rpm for 30 min to evaluate the supernatant ion concentration with ICP-MS.

#### CLSM analysis

For this experiment, the plant root tissue was treated with a 1 mL solution of CS@OA or CS@CH NPs at a concentration of 300 ppm by using a sprayer. After 1 h, the root tissue was excised from the treated plant and washed thoroughly with deionized water. Then, the root tissue was placed in an FAA fixative solution containing 37% formaldehyde (v/v), 5% glacial acetic acid (v/v), 45% ethanol (v/v) and 45% distilled water (v/v), followed by vacuum infiltration for 15 min. After that, the root tissue was transferred into a fresh FAA fixative solution overnight at 4 °C. Then, the root samples were washed three times with phosphate buffer for 10 min each. Tissues were then dehydrated through a 30, 50, 75, 90, and 100% graded series of ethanol for 15 min each. Prior to microscopy, sample clearance was performed to increase the tissue transparency. For this, tissues were passed through 25, 50, 75 and 100% concentrations of glycerol for 1 h each with two changes of each solution and then incubated overnight in pure glycerol^79^. Cleared tissues were then mounted on long coverslips in glycerol and covered by small coverslips. Images were acquired by CLSM in the blue region after excitation at 405 nm and emission was collected in the wavelength range of 410–480 nm.

#### Root tissue section preparation for TEM imaging

For this study, the plant roots were treated with 1 mL of a CS@OA or CS@CH NPs solution at a concentration of 300 ppm by using a sprayer. After 3 h, the roots were excised from the treated plants and washed thoroughly with deionized water. Then, the root samples were fixed with a 5% glutaraldehyde solution followed by incubation for 2 h at 4 °C. Then, the root samples were washed three times with phosphate-buffered saline for 10 min each time. After that, several steps, such as osmication with 1% osmium tetroxide, *en bloc* staining with 2% uranyl acetate, and dehydration with a graded series of ethanol followed by infiltration with embedding medium, were performed as reported^80^. Then, a few drops of the Spurr resin were placed into moulds, followed by transfer of the root samples into the moulds. The remaining space in the moulds was filled with Spurr resin, and then the moulds were kept overnight at 60 °C for polymerization. Then, the samples were sectioned into 70–80 nm thick samples with a Leica EM UC6 ultra-microtome, followed by staining with uranyl acetate and lead citrate. The root tissue sections were then placed on nickel grids and analyzed by TEM.

### Uptake mechanism of CS@OA and CS@CH NPs

#### AFM force measurements

The force was measured by modifying AFM silicon nitride tips with CS@OA and CS@CH NPs and fixation of fresh root tip onto a glass plate by using epoxy resin^31,59,60^. First, the glass coverslips were placed in a warm chromium acid solution for 3 h to remove residual organic matter and then rinsed with Milli-Q water followed by drying under a stream of nitrogen. AFM silicon nitride tips were modified with CS@OA and CS@CH separately, by incubation in the respective solutions for 5 h followed by sequential washing with water and ethanol. A fresh root tip was fixed onto a glass plate with an epoxy resin that could dry fast without any cross-reactions in order to keep the root alive. Following this, water was added carefully with a syringe, and the AFM tip was placed in contact with the root with the assistance of the microscope and the adhesion force was measured. The protocol we selected was to record single measurements at different points along the length of the root by taking several measurements at the root surface. AFM silicon nitride cantilevers with silicon nitride tips (type MLCT, from APP NANO) were used in all of the experiments. The spring constants of the tips were calibrated by the thermal fluctuation method and were all in the range of 0.040–0.075 N m^−1^. All of the experiments were carried out at a pulling speed of 1000 nm s^−1^. The AFM experiments were conducted after allowing the system to equilibrate for 30 min. All of the AFM force measurements were carried out at 25 ± 1 °C.

#### Protein corona study

For this study, 30-day-old plants of the same length and weight were used for each treatment. Afterwards, the plant roots were treated with 1 mL of CS@OA or CS@CH NP solutions at a concentration of 300 ppm by using a sprayer, and the plants were then transferred into a hydroponic medium for 3 h. Then, two grams of root from all the treated plants were collected and ground in liquid nitrogen with a mortar and pestle. Then, the ground tissue powder from each treatment was added to 10 mL of a 2 mM citrate buffer at pH 4.5, followed by homogenization of the samples in an ice bath. Then, a 0.5 mM phenylmethylsulphonyl fluoride (PMSF) cocktail was added to each treatment solution to inhibit protease. The optimum pH range should be 3.5–7.0 for activity of the macerozyme R-10 as provided by the manufacturer; as such, the pH of the citrate buffer was adjusted to 4.5 with citric acid. Then, 2 mL of citrate buffer containing 800 mg of the macerozyme R-10 was added to the above solution, and the samples were incubated for 24 h at 37 °C prior to being digested as reported^18^. After digestion, samples were filtered through 0.45 µm filter paper, and the filtrate was collected. Then, the samples were centrifuged at 12,000 rpm for 30 min at 4 °C to separate the NPs from unbound proteins. The obtained pellet for each treatment was washed several times by dispersing in 1.5 mL of 1 × PBS, followed by centrifugation to completely remove NPs from the unbound proteins^81^. All of the pellets (having NPs with bound proteins) had 100 µL of a 10% sodium dodecyl sulphate solution added to them and were kept at 95 °C in a water bath for 10 min, followed by centrifugation at 12,000 rpm. Then, the supernatant was collected, which had NP-free protein. After that, samples were cleaned by using a ReadyPrep 2D clean-up kit. Finally, the clean protein pellets for each treatment were used for 2D-PAGE documentation.

The cleaned sample pellets for each treatment were dispersed in 200 µL of rehydration buffer. The entire 200 μL volume of the IPG gel rehydration buffer-containing protein samples were loaded onto a 7-cm long IPG strip with a pH range of 3–10, which allows the proteins to enter the IPG strip gel after an overnight rehydration and equilibration process. After that, the IPG strips were transferred to an isoelectric focusing tray, and the proteins were separated by using an isoelectric focusing electrophoresis unit. The IPG strips were run for a total of 10 kVh. After isoelectric focusing, the IPG strips were equilibrated in equilibration buffer-I and equilibration buffer-II for 10 min each. The equilibrated IPG strips were then used for a second dimension of electrophoresis. The proteins were separated by using a 12% polyacrylamide gel. Gel electrophoresis was carried out at 100 V for approximately 80 min until the proteins had separated to the end of the gel. Precision plus protein kaleidoscope prestained protein standard was used as a molecular weight marker. The gels were stained by silver staining, and images were captured by a gel doc and analyzed by the PDQuest software (Bio-Rad).

After electrophoresis, gels were fixed in a methanol (50 mL), acetic acid (12 mL), formaldehyde (50 µL), and MQ water (38 mL) mixture for 30 min followed by washing in a methanol (20 mL) and water (80 mL) mixture for 10 min. Then, the gels were sensitized in an aqueous solution of sodium thiosulfate (prepared by adding 20 mg to 100 mL of MQ water) for 10 min followed by washing of the gels with excess MQ water. After that, the gels were stained with a chilled silver nitrate solution (prepared by adding 200 mg of silver nitrate and 76 µL of formaldehyde into 100 mL of MQ water) at 4 °C followed by washing of the gels with excess MQ water. Then, the gels were developed in a development solution (sodium thiosulfate (0.4 mg), sodium carbonate (6 g), and formaldehyde (50 µL) in 100 mL of MQ water) under a white light transilluminator. After the protein spots appeared, the development solution was replaced with the stop solution (10 mL acetic acid in 90 mL distilled water) to avoid excessive development.

#### Toxicity study

The toxicity of the hydrophobic and hydrophilic NPs was compared by using the ascorbate peroxidase (APOX) and catalase (CAT) assays as reported^51^. For this study, the tomato roots were treated with 1 mL of CS@OA or CS@CH NPs at a concentration of 300 ppm by using a sprayer as described above. For CAT activity, treated root and shoot tissues were excised after a specific time period and ground in liquid nitrogen with a mortar and pestle followed by homogenization in 1 mL of pH 7.4 ice-cold potassium phosphate buffer. Then, the tissue extracts were centrifuged at 4 °C for 5 min at 10,000 rpm, and the supernatant was collected. Then, 10 µL of the supernatant was added to 990 μL of 10 mM H_2_O_2_ in a quartz cuvette and mixed. The CAT activity was determined by a UV–Vis spectrophotometer based on the decrease in the reaction mixture absorbance at 240 nm over 1 min.

For APOX activity, plant samples were prepared as described above. A 100 μL volume of the supernatant was added to a 1 mL quartz cuvette with 886 μL of 0.1 M potassium phosphate buffer at pH 7.4, 4 μL of 25 mM ascorbate, and 10 μL of 17 mM H_2_O_2_ and then mixed. The APOX activity was determined with a UV–Vis spectrophotometer by measuring the decrease in the reaction mixture absorbance at 265 nm over one minute. The protein concentrations for both assays were quantified by the Bradford method using a bovine serum albumin standard curve.

Further, the root viability was studied by MTT assay as reported^82^. In brief, the plant roots were treated with 1 mL of CS@OA or CS@CH NPs as described above and incubated for 24 h. After that, 10 mg of the fresh root tissue was taken and transferred to the 2 mL centrifuge tube followed by the addition of MTT dye. After 4 h of incubation in the dark, the MTT solution was discarded and the root tissues were transferred to the fresh petriplates. The root tissue was cut with a sterile scalpel into 1–2 mm pieces followed by the addition of 0.5 mL of KOH solution to this. The cut root pieces along with the KOH solution was transferred to the 2 mL of centrifuge tubes and 0.5 mL of DMSO solution was added to each tube to make the total volume of 1 mL. Then, the tubes were centrifuged at room temperature at 500 × *g* for 5 min. The supernatant was transferred to fresh tubes, resultant absorbance was measured at 570 nm and cell viability was calculated.

### Statistical analysis

GraphPad Prism 8.0 software was used for all the statistical data analyses. All data were plotted as mean ± standard error. A nonparametric t-test was used to reveal the significant difference at 95% confidence level (P < 0.05), as denoted by *asterisks. The data not showing asterisks, reveal not significant.

## Supplementary information

Supplementary Information 1.

Supplementary Video 1.

Supplementary Video 2.

## References

[1] Beachy R (2002). Divergent perspectives on GM food. Nat. Biotechnol..

[2] Wang H (2018). Carbon dots promote the growth and photosynthesis of mung bean sprouts. Carbon N. Y..

[3] Giraldo JP (2014). Plant nanobionics approach to augment photosynthesis and biochemical sensing. Nat. Mater..

[4] Sonkar SK, Roy M, Babar DG, Sarkar S (2012). Water soluble carbon nano-onions from wood wool as growth promoters for gram plants. Nanoscale.

[5] Khodakovskaya MV (2011). Complex genetic, photothermal, and photoacoustic analysis of nanoparticle-plant interactions. Proc. Natl. Acad. Sci. U.S.A..

[6] Yin J, Wang Y, Gilbertson LM (2018). Opportunities to advance sustainable design of nano-enabled agriculture identified through a literature review. Environm. Sci. Nano.

[7] Koo Y (2016). In planta response of arabidopsis to photothermal impact mediated by gold nanoparticles. Small.

[8] Zhao P (2018). Translocation, distribution and degradation of prochloraz-loaded mesoporous silica nanoparticles in cucumber plants. Nanoscale.

[9] Yi Z (2015). Functionalized mesoporous silica nanoparticles with redox-responsive short-chain gatekeepers for agrochemical delivery. ACS Appl. Mater. Interfaces.

[10] Karny A, Zinger A, Kajal A, Shainsky-Roitman J, Schroeder A (2018). Therapeutic nanoparticles penetrate leaves and deliver nutrients to agricultural crops. Sci. Rep..

[11] McGehee DL, Lahiani MH, Irin F, Green MJ, Khodakovskaya MV (2017). Multiwalled carbon nanotubes dramatically affect the fruit metabolome of exposed tomato plants. ACS Appl. Mater. Interfaces.

[12] Liu Q (2010). Study of the inhibitory effect of water-soluble fullerenes on plant growth at the cellular level. ACS Nano.

[13] Wang Y (2017). Agglomeration determines effects of carbonaceous nanomaterials on soybean nodulation, dinitrogen fixation potential, and growth in soil. ACS Nano.

[14] da Cruz TNM (2019). A new glance on root-to-shoot in vivo zinc transport and time-dependent physiological effects of ZnSO4 and ZnO nanoparticles on plants. Sci. Rep..

[15] Zhao J (2017). Uptake, distribution, and transformation of CuO NPs in a floating plant eichhornia crassipes and related stomatal responses. Environ. Sci. Technol..

[16] Marshall CE, Upchurch WJ (1953). Free energy in cation interchange as illustrated by plant root-substrate relationships. J. Phys. Chem..

[17] Dan Y (2015). Characterization of gold nanoparticle uptake by tomato plants using enzymatic extraction followed by single-particle inductively coupled plasma-mass spectrometry analysis. Environ. Sci. Technol..

[18] Jiménez-Lamana J, Wojcieszek J, Jakubiak M, Asztemborska M, Szpunar J (2016). Single particle ICP-MS characterization of platinum nanoparticles uptake and bioaccumulation by *Lepidium sativum* and *Sinapis alba* plants. J. Anal. At. Spectrom..

[19] Schymura S, Fricke T, Hildebrand H, Franke K (2017). Elucidating the role of dissolution in CeO _2_ nanoparticle plant uptake by smart radiolabeling. Angew. Chemie Int. Ed..

[20] Peng C (2017). Fate and transformation of CuO nanoparticles in the soil-rice system during the life cycle of rice plants. Environ. Sci. Technol..

[21] Stegemeier JP (2015). Speciation Matters: Bioavailability of silver and silver sulfide nanoparticles to alfalfa (*Medicago sativa* ). Environ. Sci. Technol..

[22] del Pradas Real AE (2017). Silver nanoparticles and wheat roots: A complex interplay. Environ. Sci. Technol..

[23] Serag MF (2011). Functional platform for controlled subcellular distribution of carbon nanotubes. ACS Nano.

[24] Wang Z (2012). Xylem- and phloem-based transport of CuO nanoparticles in maize (Zea mays L.). Environ. Sci. Technol..

[25] Barrios AC (2016). Effects of uncoated and citric acid coated cerium oxide nanoparticles, bulk cerium oxide, cerium acetate, and citric acid on tomato plants. Sci. Total Environ..

[26] Wong MH (2016). Lipid exchange envelope penetration (LEEP) of nanoparticles for plant engineering: A universal localization mechanism. Nano Lett..

[27] Sharma S (2020). Effect of galvanotaxic graphene oxide on chloroplast activity: Interaction quantified with Biolayer-Interferometry coupled confocal microscopy. Carbon N. Y..

[28] Torney F, Trewyn BG, Lin VSY, Wang K (2007). Mesoporous silica nanoparticles deliver DNA and chemicals into plants. Nat. Nanotechnol..

[29] Vijayakumar PS, Abhilash OU, Khan BM, Prasad BLV (2010). Nanogold-loaded sharp-edged carbon bullets as plant-gene carriers. Adv. Funct. Mater..

[30] Winkleman A, Gotesman G, Yoffe A, Naaman R (2008). Immobilizing a drop of water: Fabricating highly hydrophobic surfaces that pin water droplets. Nano Lett..

[31] Dague E (2007). Chemical force microscopy of single live cells. Nano Lett..

[32] Wertz CF, Santore MM (2001). Effect of surface hydrophobicity on adsorption and relaxation kinetics of albumin and fibrinogen: Single-species and competitive behavior. Langmuir.

[33] Jiang W (2017). Comparison study on four biodegradable polymer coatings for controlling magnesium degradation and human endothelial cell adhesion and spreading. ACS Biomater. Sci. Eng..

[34] Hasan A, Pattanayek SK, Pandey LM (2018). Effect of functional groups of self-assembled monolayers on protein adsorption and initial cell adhesion. ACS Biomater. Sci. Eng..

[35] Rasch MR (2010). Hydrophobic gold nanoparticle self-assembly with phosphatidylcholine lipid: Membrane-loaded and janus vesicles. Nano Lett..

[36] Ungati H, Govindaraj V, Narayanan M, Mugesh G (2019). Probing the formation of a seleninic acid in living cells by the fluorescence switching of a glutathione peroxidase mimetic. Angew. Chemie - Int. Ed..

[37] Frankenberger WT, Engberg RA, Engberg RA (1998). Effects of Selenium Supplementation of Fertilizers on Human Nutrition and Selenium Status. Environmental Chemistry of Selenium.

[38] Pedrero Z, Madrid Y, Hartikainen H, Cámara C (2008). Protective effect of selenium in Broccoli ( Brassica oleracea) plants subjected to cadmium exposure. J. Agric. Food Chem..

[39] Levard C (2013). Sulfidation of silver nanoparticles: Natural antidote to their toxicity. Environ. Sci. Technol..

[40] Wang Z, Zhang L, Zhao J, Xing B (2016). Environmental processes and toxicity of metallic nanoparticles in aquatic systems as affected by natural organic matter. Environ. Sci. Nano.

[41] Teotia RS (2015). Bifunctional polysulfone-chitosan composite hollow fiber membrane for bioartificial liver. ACS Biomater. Sci. Eng..

[42] Lau C, Cooney MJ, Atanassov P (2008). Conductive macroporous composite chitosan−carbon nanotube scaffolds. Langmuir.

[43] Arakha M (2015). Antimicrobial activity of iron oxide nanoparticle upon modulation of nanoparticle-bacteria interface. Sci. Rep..

[44] Phan CM, Nguyen HM (2017). Role of capping agent in wet synthesis of nanoparticles. J. Phys. Chem. A.

[45] USDA Economics, Statistics and Market Information System. Available at: https://usda.library.cornell.edu/. (Accessed: 12th November 2019)

[46] Schilmiller AL, Howe GA (2005). Systemic signaling in the wound response. Curr. Opin. Plant Biol..

[47] Vijayakumar PS, Selvakumar S, Gholap RS, Singh AP, Prasad BLV (2010). Vice to virtue: Intracellular biogenic nanoparticles for the generation of carbon supported catalysts. J. Nanosci. Nanotechnol..

[48] Anicuta, S., Dobre, L.-M., Stroescu, M. & Jipa, I. Fourier transform infrared (FTIR) spectroscopy for characterization of antimicrobial films containing chitosan. *Analele Universitatii din Oradea fascicula: Ecotoxicologie, Zootehniesitehnologii de Industrie Alimentara* 1234–1240 (2010).

[49] Li WP (2013). Eccentric inorganic-polymeric nanoparticles formation by thermal induced cross-linked esterification and conversion of eccentricity to raspberry-like Janus. Chem. Commun..

[50] Zhi J, Zhang LZ (2017). Durable superhydrophobic surfaces made by intensely connecting a bipolar top layer to the substrate with a middle connecting layer. Sci. Rep..

[51] Hernandez-Viezcas JA, Castillo-Michel H, Peralta-Videa JR, Gardea-Torresdey JL (2016). Interactions between CeO_2_ nanoparticles and the desert plant mesquite: A spectroscopy approach. ACS Sustain. Chem. Eng..

[52] Hu T (2018). Absorption and bio-transformation of selenium nanoparticles by wheat seedlings (Triticum aestivum L.). Front. Plant Sci..

[53] Dwivedi AD (2018). Uptake, distribution, and transformation of zerovalent iron nanoparticles in the edible plant *Cucumis sativus*. Environ. Sci. Technol..

[54] Ristroph KD, Astete CE, Bodoki E, Sabliov CM (2017). Zein nanoparticles uptake by hydroponically grown soybean plants. Environ. Sci. Technol..

[55] Parsons JG, Lopez ML, Gonzalez CM, Peralta-Videa JR, Gardea-Torresdey JL (2010). Toxicity and biotransformation of uncoated and coated nickel hydroxide nanoparticles on mesquite plants. Environ. Toxicol. Chem..

[56] Priester JH (2012). Soybean susceptibility to manufactured nanomaterials with evidence for food quality and soil fertility interruption. Proc. Natl. Acad. Sci. USA.

[57] Liu Q (2009). Carbon nanotubes as molecular transporters for walled plant cells. Nano Lett..

[58] Serag MF (2011). Trafficking and subcellular localization of multiwalled carbon nanotubes in plant cells. ACS Nano.

[59] Fernandes AN (2012). Mechanical properties of epidermal cells of whole living roots of *Arabidopsis thaliana*: An atomic force microscopy study. Phys. Rev. E Stat. Nonlinear Soft Matter. Phys..

[60] Dufrêne YF, Martínez-Martín D, Medalsy I, Alsteens D, Müller DJ (2013). Multiparametric imaging of biological systems by force-distance curve-based AFM. Nat. Methods.

[61] Javier AM (2006). Combined atomic force microscopy and optical microscopy measurements as a method to investigate particle uptake by cells. Small.

[62] Zyuzin MV (2017). Role of the protein corona derived from human plasma in cellular interactions between nanoporous human serum albumin particles and endothelial cells. Bioconjug. Chem..

[63] Huang H (2016). An evaluation of blood compatibility of silver nanoparticles. Sci. Rep..

[64] Sakulkhu U, Mahmoudi M, Maurizi L, Salaklang J, Hofmann H (2014). Protein corona composition of superparamagnetic iron oxide nanoparticles with various physico-chemical properties and coatings. Sci. Rep..

[65] Li Y (2018). Protein corona of airborne nanoscale PM2.5 induces aberrant proliferation of human lung fibroblasts based on a 3D organotypic culture. Sci. Rep..

[66] Ke PC, Lin S, Parak WJ, Davis TP, Caruso F (2017). A decade of the protein corona. ACS Nano.

[67] Pino PD (2014). Protein corona formation around nanoparticles—From the past to the future. Mater. Horizons.

[68] Benabdellah K, Azcon-Aguilar C, Ferrol N (1998). Soluble and membrane symbiosis-related polypeptides associated with the development of arbuscular mycorrhizas in tomato (*Lycopersicon esculentum*). New Phytol..

[69] Kaveh R (2013). Changes in *Arabidopsis thaliana* gene expression in response to silver nanoparticles and silver ions. Environ. Sci. Technol..

[70] Turakainen M, Hartikainen H, Seppänen MM (2004). Effects of selenium treatments on potato (*Solanum tuberosum* L.) growth and concentrations of soluble sugars and starch. J. Agric. Food Chem..

[71] Xue T, Hartikainen H, Piironen V (2001). Antioxidative and growth-promoting effect of selenium on senescing lettuce. Plant Soil.

[72] Sun X, Zhong Y, Huang Z, Yang Y (2014). Selenium accumulation in unicellular green alga chlorella vulgaris and its effects on antioxidant enzymes and content of photosynthetic pigments. PLoS ONE.

[73] Seppänen M, Turakainen M, Hartikainen H (2003). Selenium effects on oxidative stress in potato. Plant Sci..

[74] Hartikainen H, Xue T, Piironen V (2000). Selenium as an anti-oxidant and pro-oxidant in ryegrass. Plant Soil.

[75] Sharma S, Singh S, Ganguli AK, Shanmugam V (2017). Anti-drift nano-stickers made of graphene oxide for targeted pesticide delivery and crop pest control. Carbon N. Y..

[76] Roy Chowdhury S (2018). Remarkably efficient blood-brain barrier crossing polyfluorene-chitosan nanoparticle selectively tweaks amyloid oligomer in cerebrospinal fluid and Aβ1–40. ACS Omega.

[77] Salehi H, Chehregani A, Lucini L, Majd A, Gholami M (2018). Morphological, proteomic and metabolomic insight into the effect of cerium dioxide nanoparticles to *Phaseolus vulgaris* L. under soil or foliar application. Sci. Total Environ..

[78] Jarstfer AG, Farmer-Koppenol P, Sylvia DM (1998). Tissue magnesium and calcium affect arbuscular mycorrhiza development and fungal reproduction. Mycorrhiza.

[79] Kitin P, Funada R, Sano Y, Ohtani J (2000). Analysis by confocal microscopy of the structure of cambium in the hardwood Kalopanax pictus. Ann. Bot..

[80] Wu S, Baskin TI, Gallagher KL (2012). Mechanical fixation techniques for processing and orienting delicate samples, such as the root of *Arabidopsis thaliana*, for light or electron microscopy. Nat. Protoc..

[81] Dobrovolskaia MA (2009). Interaction of colloidal gold nanoparticles with human blood: Effects on particle size and analysis of plasma protein binding profiles. Nanomed. Nanotechnol. Biol. Med..

[82] Majumdar S, Guha T, Kundu R (2017). MTT assay for cytotoxicity assessment in oryza sativa root tissue. Bio-Protocol.

