# Supplementary material for: A non-classical route of efficient plant uptake verified with fluorescent nanoparticles and root adhesion forces investigated using AFM
Source: Sci Rep. 2020 Nov 6;10:19233. doi: 10.1038/s41598-020-75685-3 (PMC7648022; doi:10.1038/s41598-020-75685-3)
Supplement: Supplementary file 1 — Supplementary Information 1. [file 41598_2020_75685_MOESM1_ESM.docx]

Supplementary Information

**A non-classical route of efficient plant uptake verified with fluorescent nanoparticles and root adhesion forces investigated using AFM**

Sandeep Sharma^1^, Mohd. Muddassir^2^, Saraladevi Muthusamy^3^, Pardeep Kumar Vaishnav^4^, Manish Singh^1^, Deepak Sharma^2^, Selvaraju Kanagarajan^5^* and Vijayakumar Shanmugam^1^*

**^1^**Institute of Nano Science and Technology, Habitat Centre, Phase- 10, Sector- 64, Mohali, Punjab – 160062, India.

**^2^**CSIR-Institute of Microbial Technology, Chandigarh, India.

**^3^**Applied Microbiology, Department of Chemistry, Lund University, Lund, Sweden.

^4^Microscopy division, AIIMS, New Delhi.

^5^Department of Plant Breeding, Swedish University of Agricultural Sciences, Alnarp, Sweden.

***Corresponding Authors**

Selvaraju Kanagarajan ([selvaraju.kanagarajan@slu.se](mailto:selvaraju.kanagarajan@slu.se)); Vijayakumar Shanmugam ([vijayakumarshanmugham@gmail.com](mailto:vijayakumarshanmugham@gmail.com)).


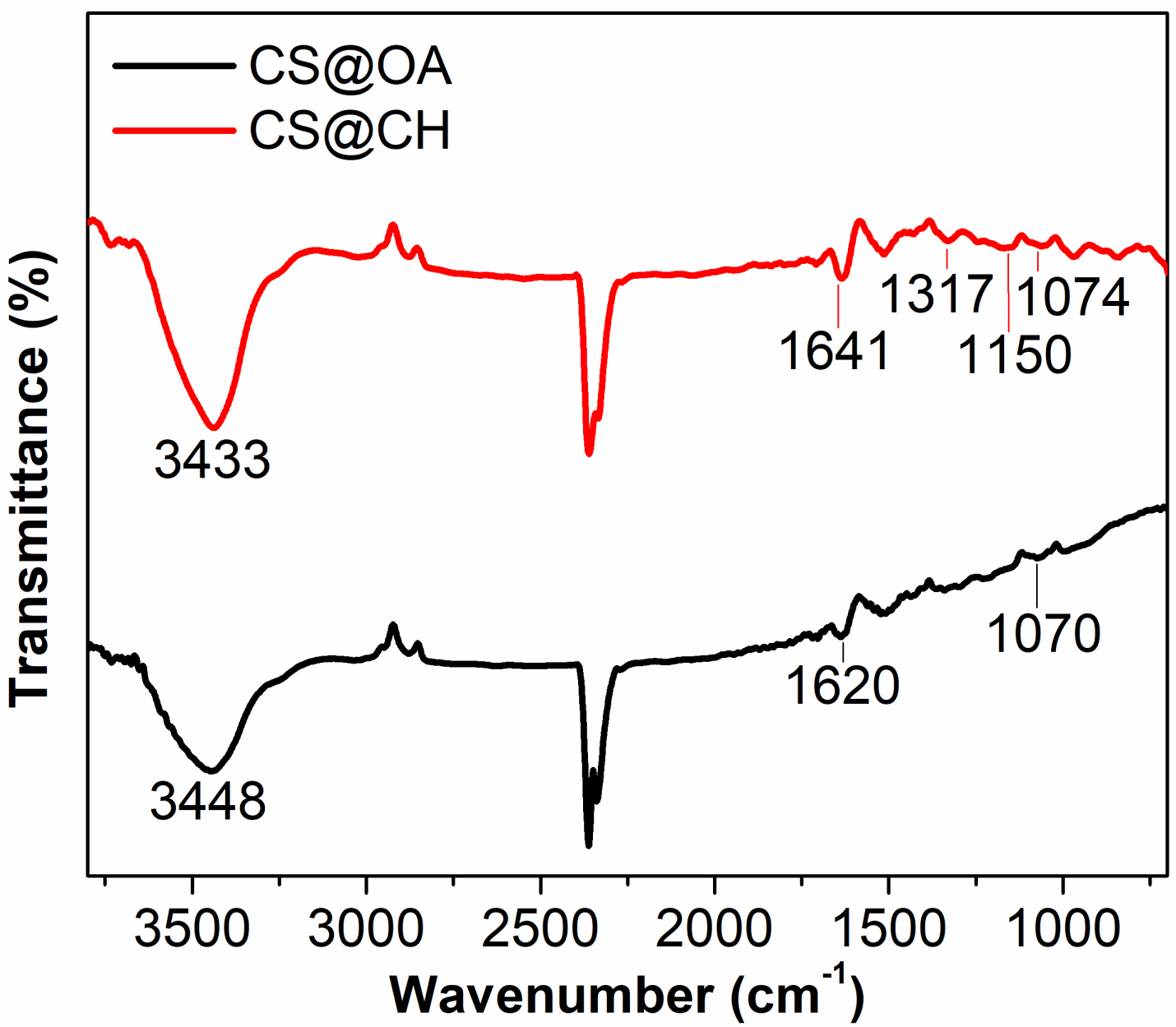


Figure S1**.** FT-IR spectra of CS@OA and CS@CH NPs**.**


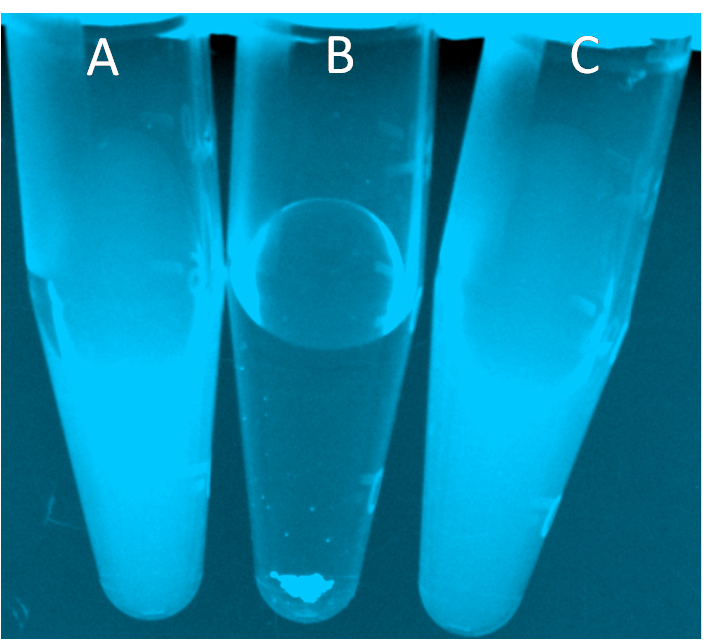


Figure S2. Fluorescent images of; (A) dispersion of CS@OA NPs in ethanol, (B) aggregation of hydrophobic CS@OA NPs in distilled water, (C) dispersion of CS@CH NPs in distilled water.


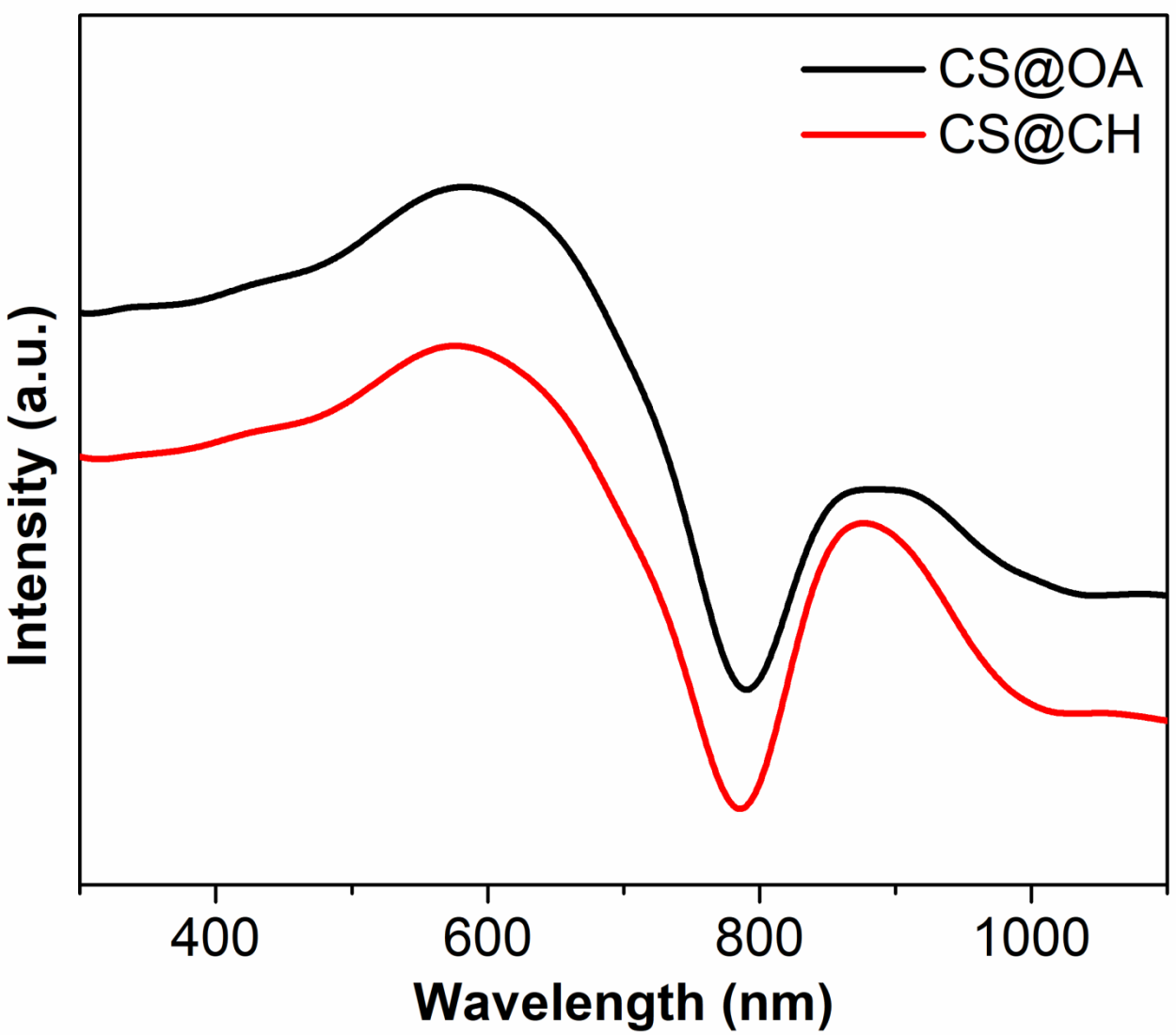


Figure S3. UV-Visible spectra of CS@OA and CS@CH in 1:1 ratio of ethanol/water mixture.


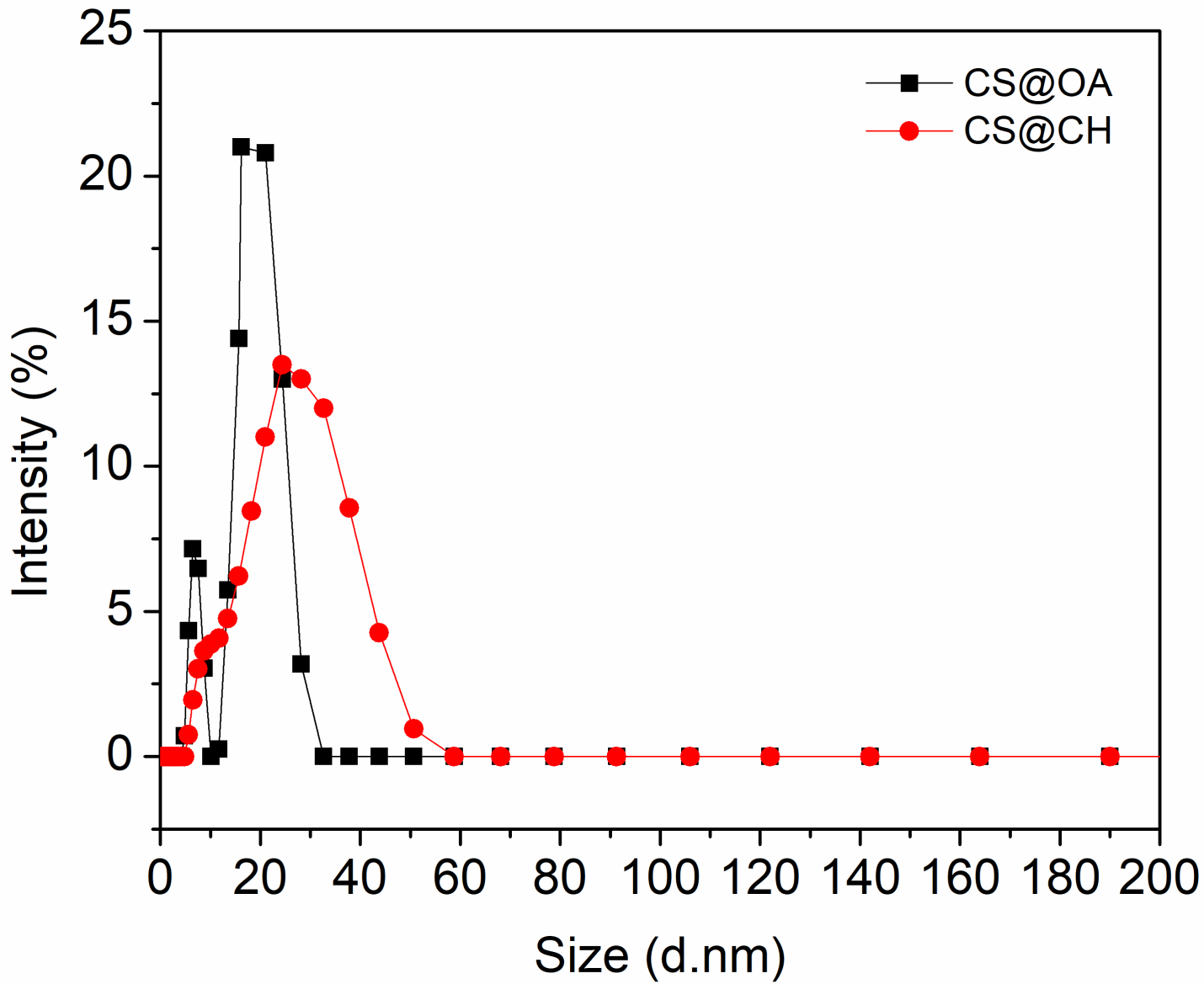


Figure S4. DLS of CS@OA and CS@CH NPs.


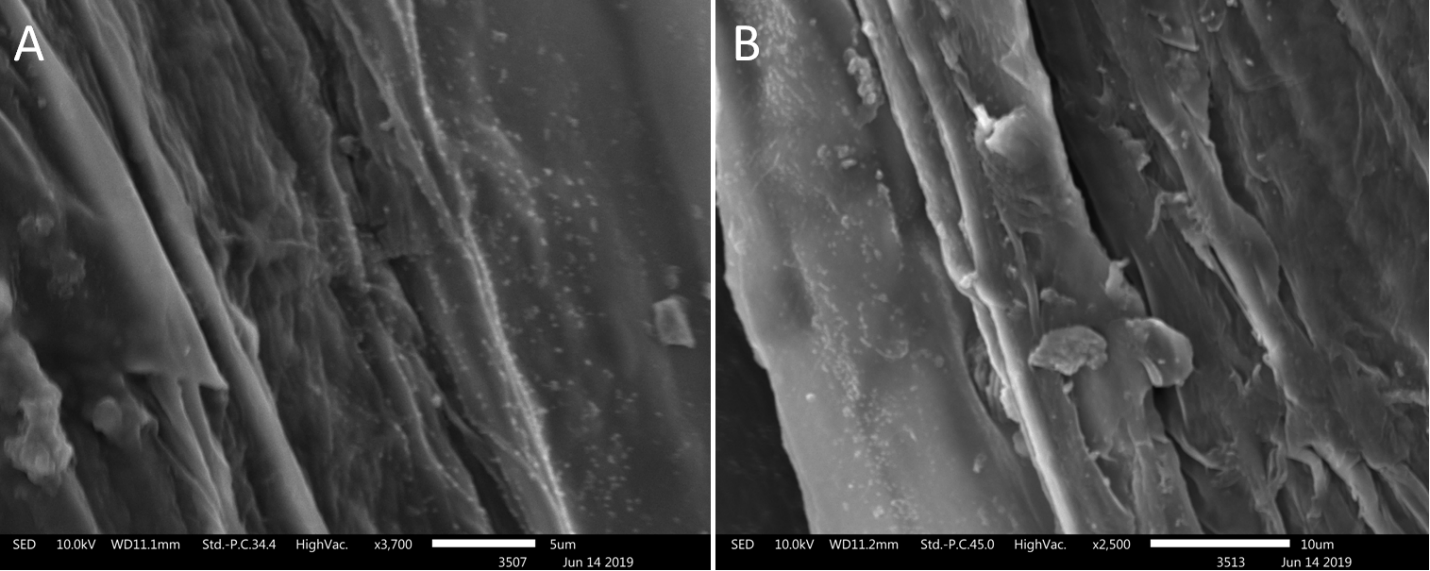


Figure S5. SEM image of the roots not washed with 0.1M HNO_3_ after incubation with (A) CS@OA and (B) CS@CH NPs.


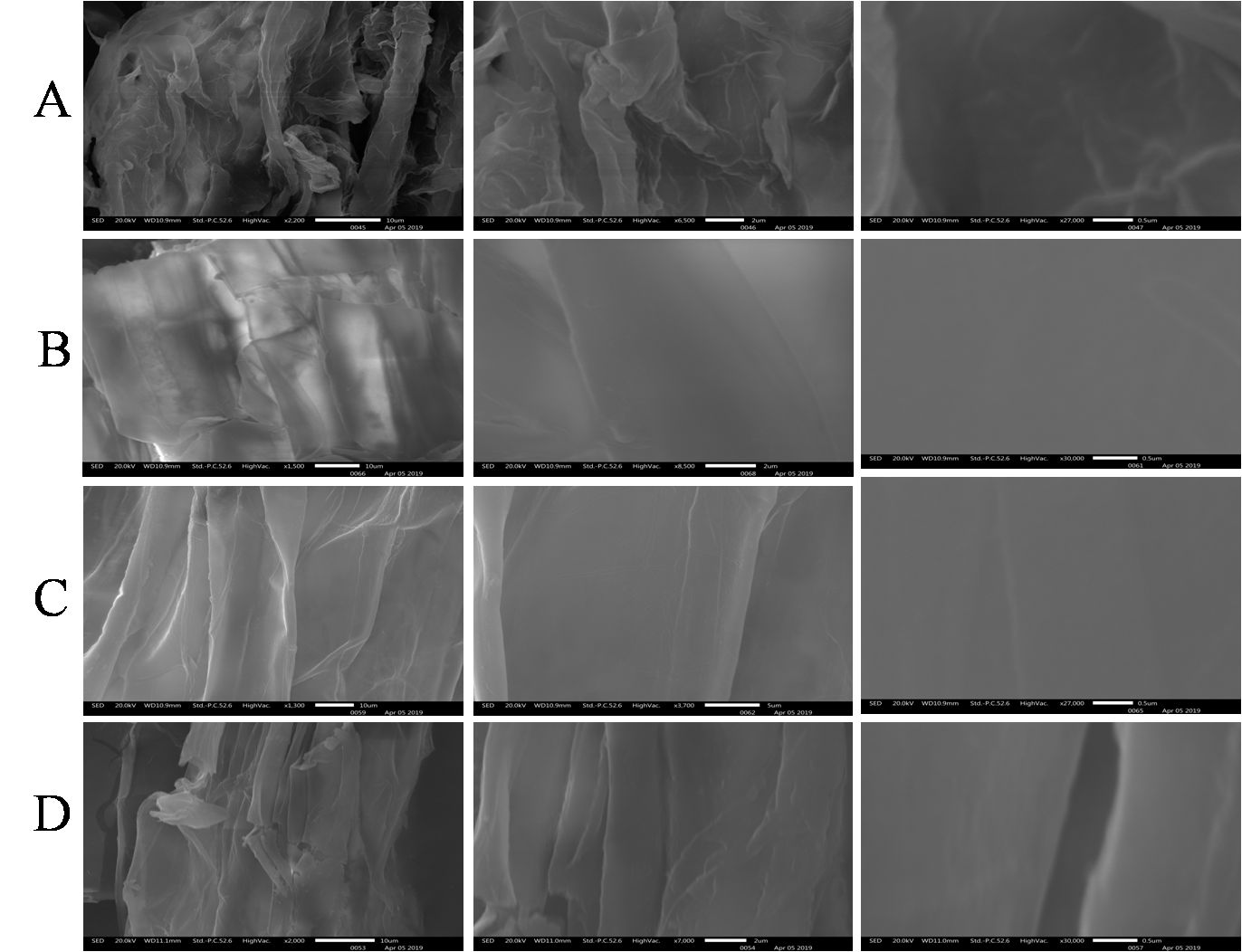


Figure S6. SEM image of; (A, B) Root washed with 0.1M HNO_3_ after incubation with CS@OA and (C, D) CS@CH NPs (Images are capture at two different area and different scale bar of 10 µm, 2 µm and 500 nm).


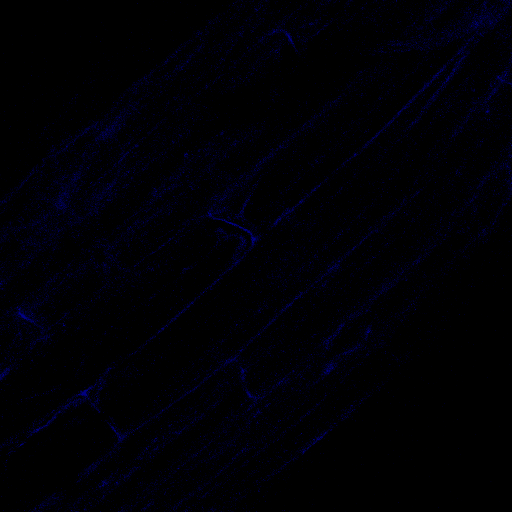


Figure S7. CLSM image of untreated tomato root. The image after intense scanning shows blue fluorescence grains spread all over, without location specificity. Thus, this is not from the particles but by the auto-fluorescence from the plant tissue.


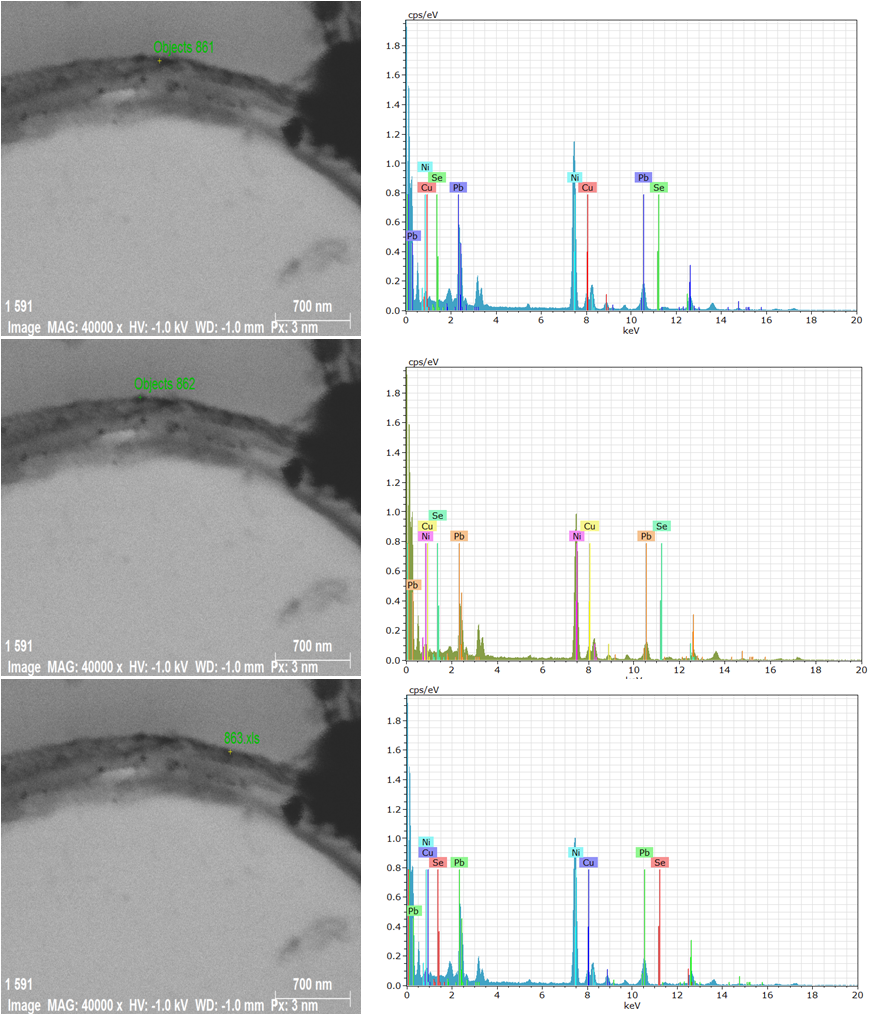


Figure S8. Point EDX performed on various NPs showing the signal for copper.


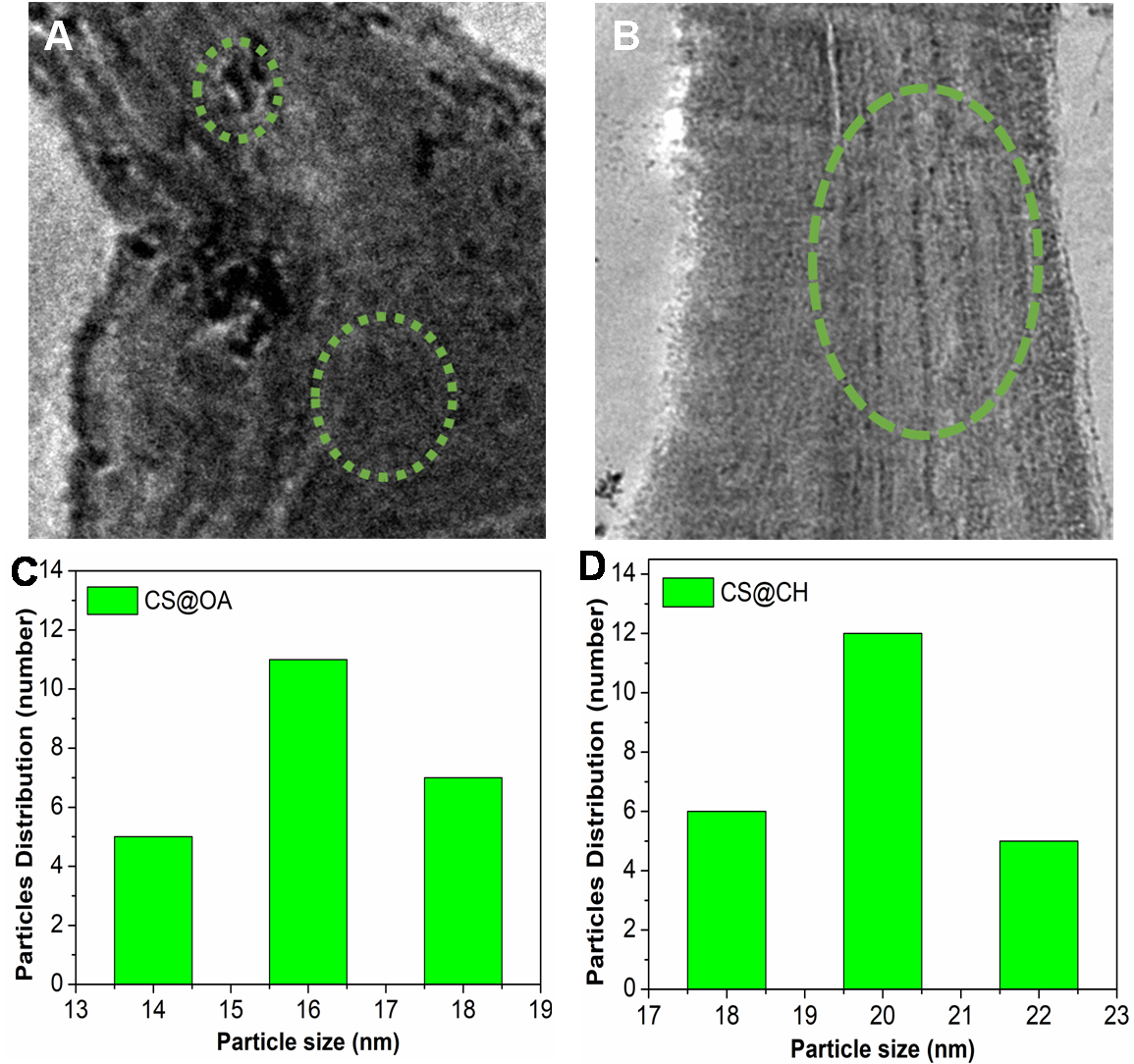


Figure S9. Particle size distribution of CS@OA and CS@CH NPs in plant root tissue.


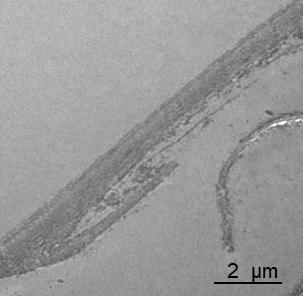


Figure S10. TEM image of untreated tomato root.


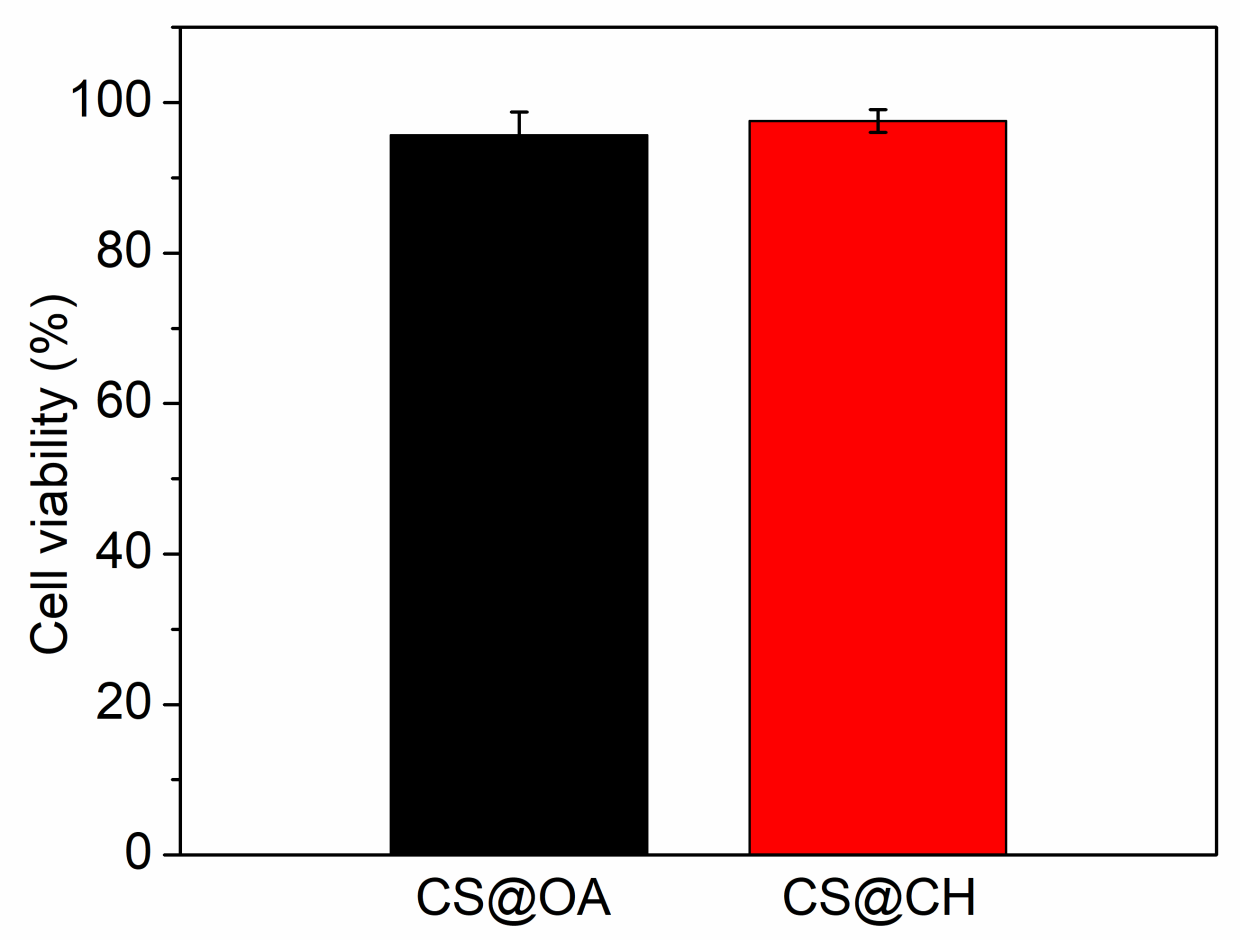


Figure S11. The MTT assay showing the root viability after 24 h of treatment with CS@OA and CS@CH NPs.
